# Quantitative Assessment of Randomized DNA Base Sequences Using Multi‐Model Physical Analysis for High‐Fidelity Data Storage

**DOI:** 10.1002/advs.202517208

**Published:** 2025-11-21

**Authors:** Seongjun Seo, Thi Hong Nhung Vu, Anshula Tandon, Suyoun Park, Thi Bich Ngoc Nguyen, Shinsuke Kawai, Sung Ha Park

**Affiliations:** ^1^ Department of Physics Institute of Basic Science and Sungkyunkwan Advanced Institute of Nanotechnology (SAINT) Sungkyunkwan University Suwon 16419 Republic of Korea; ^2^ Faculty of Science Yamagata University Yamagata 990‐8560 Japan

**Keywords:** 3‐input 1‐output logic algorithm, active particle trajectory model, DNA data storage, inverse Ising model, sequence randomness

## Abstract

DNA is emerging as a promising medium for ultra‐dense, long‐term digital data storage, yet sequence design remains hindered by homopolymer formation and compositional bias, which compromise synthesis, sequencing, and decoding accuracy. Here, the study introduces a quantitative framework to evaluate and optimize randomized DNA base sequence design rules using three physics‐inspired models: translational and rotational active particle trajectories, the inverse Ising model, and a 3‐input 1‐output logic algorithm system. Encoding schemes with varying homopolymer constraints are systematically applied to binary image data. Rigorous analysis reveals that stringent randomization rules markedly reduce homopolymer length, balance GC content, and enhance sequence randomness. Experimental validation via polymerase chain reaction (PCR) amplification and Sanger sequencing confirms high decoding fidelity (95–98%). This multi‐model assessment establishes a robust strategy for designing DNA sequences with superior stability, reliability, and scalability for future molecular data storage systems.

## Graphical abstract

This study quantitatively evaluates randomized DNA sequence design for digital data storage using three physics‐based models. By applying encoding schemes with strict homopolymer constraints, the framework improves base randomness, GC balance, and error resilience. Experimental validation via PCR and Sanger sequencing confirms 95–98% decoding accuracy, demonstrating the feasibility of robust, long‐term molecular information preservation.

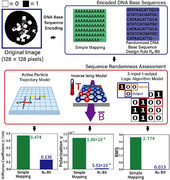

## 1 Introduction

DNA has emerged as a compelling medium for next‐generation digital data storage, offering unmatched information density, chemical stability over millennia, and the capacity for massive parallelism in synthesis and sequencing processes. With a theoretical storage density exceeding 10^18^ bytes per gram, DNA significantly outperforms conventional magnetic and solid‐state storage media. This extraordinary potential has inspired diverse encoding strategies that successfully translate text, images, audio, and video into synthetic DNA sequences, demonstrating proof‐of‐concept systems with remarkable data retention and retrieval capabilities.^[^ ^1^, ^2^, ^3^, ^4^, ^5^, ^6^, ^7^ ^]^

Despite these advancements, several key challenges hinder the practical implementation of DNA‐based storage systems.^[^ ^8^, ^9^, ^10^, ^11^, ^12^, ^13^, ^14^ ^]^ The biochemical synthesis of long DNA strands is prone to substitution, insertion, and deletion errors, while sequencing processes introduce additional inaccuracies. These errors are often exacerbated by intrinsic sequence features—extended homopolymer runs, skewed GC content, and repetitive motifs—that destabilize the molecule during synthesis, reduce sequencing accuracy, and complicate decoding. Such sequence‐dependent biases can lead to uneven amplification, base‐calling errors, and loss of data integrity over time.

To address such limitations, a variety of coding schemes have been proposed to constrain sequence composition and repetition length. These range from simple mapping methods to more complex algorithms that incorporate run‐length limits, GC balancing, and error‐correcting redundancy. While these strategies reduce bias and improve biochemical handling, their design has largely relied on heuristic rules rather than systematic, quantitative evaluation. This has resulted in a lack of consensus on optimal sequence design principles for different storage scenarios, particularly when balancing information density against biochemical robustness. Recently, several systematic and data‐driven frameworks have been introduced to overcome these issues, including graph‐based constraint encoding, deep‐learning‐assisted DNA storage, and combinatorial shortmer encoding. In addition, recent studies such as HELIX for biomedical image storage and DNA StairLoop for high‐fidelity error correction further demonstrate the rapid development of data‐aware and error‐resilient DNA storage models.^[^ ^15^, ^16^, ^17^, ^18^, ^19^ ^]^

Assessing sequence randomness—defined here as the absence of structural bias at multiple scales—is central to evaluating the effectiveness of DNA encoding schemes. Randomness influences both physical properties, such as hybridization stability and folding propensity, and functional outcomes, including synthesis efficiency, amplification uniformity, and sequencing fidelity. However, most existing assessments employ purely statistical descriptors that fail to capture the physical or structural consequences of specific sequence patterns. A deeper understanding of how base‐level constraints manifest in measurable physical parameters is essential for guiding encoding strategies.

In this context, there is a need for an evaluation framework that bridges statistical randomness metrics with physical models of sequence behaviour. Such a framework should accommodate diverse encoding schemes, quantify their effects across multiple independent measures, and be applicable to realistic digital data inputs, enabling a more principled approach to molecular data storage design.

Herein, we introduce a physics‐inspired framework for the quantitative assessment of randomized DNA base sequence design rules, integrating three complementary models: i) translational and rotational active particle trajectory analysis,^[^ ^20^, ^21^, ^22^ ^]^ ii) inverse Ising spin‐interaction modeling,^[^ ^23^, ^24^, ^25^, ^26^ ^]^ and iii) a 3‐input 1‐output logic algorithm system.^[^ ^27^, ^28^, ^29^, ^30^, ^31^, ^32^ ^]^ This approach enables multi‐scale evaluation of sequence randomness and compositional balance under varying homopolymer constraints, applied to binary image datasets as representative digital information. The framework is designed to provide an interpretable connection between algorithmic encoding rules and the physical characteristics of the resulting DNA sequences, offering a versatile tool for the rational design of high‐quality molecular data storage codes.

## 2 Results and Discussion

### 2.1 Quantitative Randomness Assessment of Encoded DNA Base Sequences Using Three Physics‐Based Models

**Figure 1:**
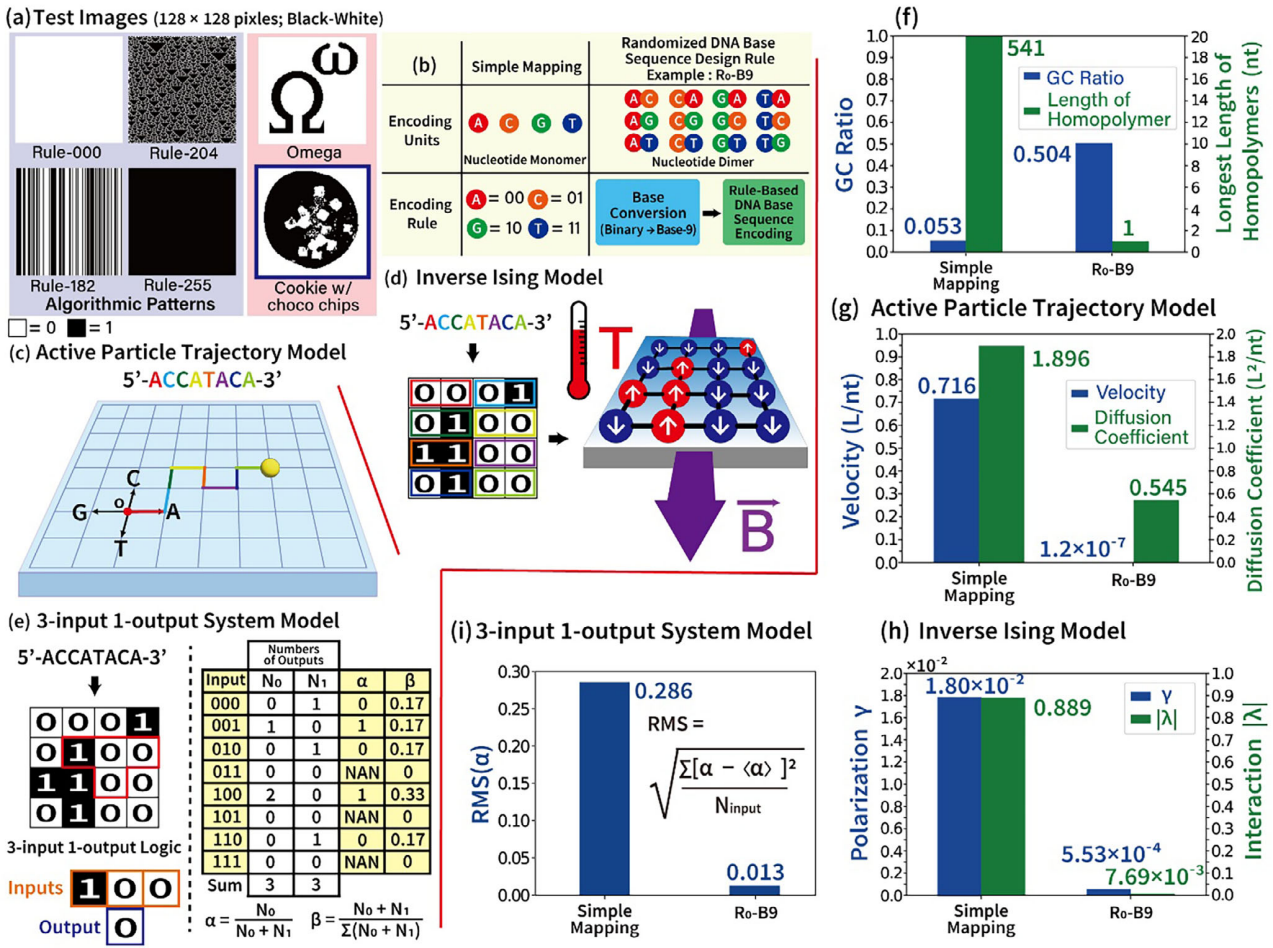
Schematic illustration of three physics‐based models for assessing sequence randomness: active particle trajectory (aPT), inverse Ising (iIsing), and 3‐input 1‐output logic algorithm system (3‐1 LA), along with their representative results. a) Six test images (128 × 128 pixels, black and white) used as original binary data. Four algorithmic pattern images are generated using the 3‐input 1‐output logic rules (i.e., Rule‐000, Rule‐182, Rule‐204, and Rule‐255). A Cookie image is created by converting an RGB image to black and white through down‐sampling. b) Characteristics of simple mapping and randomized DNA base sequence design rules, such as R_0_‐B9. In simple mapping, two‐bit binary pairs are converted directly to nucleotides (00 to A, 01 to C, 10 to G, 11 to T). The R_0_‐B9 rule, on the other hand, involves converting binary strings to Base‐9 strings before encoding them into DNA sequences. c–e) Schematic depictions of the three models for randomness assessment. The aPT, iIsing, and 3‐1 LA models use Brownian motion, binary spin alignment, and logic rules for randomization assessments, respectively. In both the iIsing and 3‐1 LA models, DNA base sequences are converted into binary strings, which are then arranged into 2D matrices. f) Sequence characteristics such as GC ratio and the longest length of homopolymers in encoded DNA base sequences. g–i) Representative results of sequence randomness assessment from applying the three models to the Cookie image. Randomness is assessed using velocity and diffusion coefficients in the aPT model, polarization factor, and interaction magnitude in the iIsing model, and root mean square of the ratio of zero‐bit outputs to total outputs in the 3‐1 LA model. [Statistical analysis; data sample size in (f–i) is one (n = 1).]

**Figure** **1**a presents six original test images (128 × 128 pixels, Black‐White) used as the source for binary data encoding into DNA base sequences. Four algorithmically generated patterns in these test images are produced using 3‐input 1‐output logic rules (Rule‐000, Rule‐182, Rule‐204, and Rule‐255). Additionally, the Omega and Cookie images are created by manually writing and down‐sampling RGB photographic images into Black‐White pixel images, respectively.

Figure 1b highlights the characteristics of two representative DNA encoding schemes: simple mapping and randomized DNA base sequence design rules (denoted as R_N_‐B#, e.g., R_0_‐B9). In a randomized DNA base sequence design rule, the subscript N in R_N_ specifies the maximum allowable homopolymer length (e.g., ∞, 2, 1, or 0), as constrained by the given randomized DNA base sequence design rule. The numerical base system required to encode binary data depends on the number of encoding units (nucleotide dimers) available in each randomized rule. A Black–White binary image can be encoded into a DNA base sequence using a randomized design rule. For example, the R_0_‐B9 design rule encodes binary strings into DNA base sequences that do not contain homopolymers (indicated by R_0_ in R_0_‐B9) by converting binary strings into Base‐9 numeral strings (indicated by B9 in R_0_‐B9) through rule‐based encoding. Following this rule, the R_0_‐B9‐based DNA sequence is generated by converting each digit in the Base‐9 numeral string into nucleotide dimer encoding units (e.g., AC, AG, AT, TA) according to the R_0_‐B9 encoding rule (see Table S1, Supporting Information). In contrast, the simple mapping method encodes two binary bits directly into single nucleotide bases (e.g., 00 → A, 01 → C, 10 → G, 11 → T).

Figure 1c–e present schematic representations of three physics‐based models used to assess the randomness of DNA base sequences: the active particle trajectory (aPT), the inverse Ising (iIsing), and the 3‐input 1‐output logic rule models. The active particle trajectory model (Figure 1c) simulates the motion of self‐propelled particles (e.g., bacteria or Janus particles) to evaluate DNA base sequence randomness by analysing their displacement at each time step (1‐nt) in 2D space. Each nucleotide base corresponds to a unit vector in one of four directions: A = (L, 0), C = (0, L), G = (−L, 0), and T = (0, −L), where L represents the unit step length. Randomness is quantified by calculating the velocity (*V*), which indicates directional bias, and the diffusion coefficient (*D*), which measures the degree of sequence randomness, based on the mean square displacement (MSD) of the trajectory. The inverse Ising model (Figure 1d) assesses randomness by calculating three parameters: the polarization factor (γ), interaction strength factor (λ), and bias factor (*h*), derived from the arrangement of up‐spins (1 bits) and down‐spins (0 bits) in a 2D lattice. The lattice is constructed by converting the DNA base sequence into a binary string via simple mapping (e.g., A → 00, C → 01, G → 10, and T → 11) and reshaping the binary string into a 2D matrix. These parameters reflect the underlying randomness or structure of the sequence. The 3‐input 1‐output logic algorithm system (Figure 1e) evaluates randomness by analyzing the output distributions of all possible 3‐bit inputs (i.e., 000, 001, 010, 011, 100, 101, 110, and 111) within a binary matrix. The randomness is quantified by computing the ratio of zero‐bit outputs to total outputs (α) and the input distribution ratio (β) for each input combination.

Figure 1f–i present sample results of sequence randomness assessments (encoded using simple mapping and R_0_‐B9) applied to the Cookie image, analyzed through the three physics‐based models. Figure 1f highlights the sequence characteristics, such as GC ratio and longest homopolymer length, in the encoded DNA base sequences. The sequence encoded using simple mapping exhibits a biased GC ratio and an extremely long homopolymer length (exceeding 500‐nt). In contrast, the sequence encoded using R_0_‐B9 maintains a balanced GC ratio and a fixed 1‐nt homopolymer length (i.e., non‐homopolymer) due to the sequence repetition constraints. Figure 1g shows randomness assessment results from the active particle trajectory model. Compared to the simple mapping‐encoded sequence, the DNA base sequence encoded using R_0_‐B9 demonstrates reduced velocity (*V*), which is proportional to homopolymer length (lower velocity indicates higher randomness). Additionally, the diffusion coefficient (*D*), a measure of sequence randomness (lower values indicate relatively better randomness), is reduced in the R_0_‐B9‐encoded sequence. Figure 1h illustrates the randomness evaluation results from the inverse Ising model, displaying two parameters: the polarization factor (γ) and the magnitude of the interaction strength factor (|λ|). The lattice structure derived from the more randomized R_0_‐B9‐encoded DNA base sequence exhibits weaker γ and |λ| values compared to the lattice generated from the simple mapping‐encoded sequence, reflecting improved randomness in the R_0_‐B9 encoding. Figure 1i presents the assessment results using the 3‐input 1‐output system model. The root mean square (RMS) of the ratio of zero‐bit outputs to total outputs (α) is shown. The binary matrix derived from the R_0_‐B9‐encoded DNA base sequence exhibits a more unbiased and evenly distributed arrangement of 0s and 1s compared to the matrix generated by simple mapping. This results in a significantly lower RMS value for the R_0_‐B9‐encoded sequence, indicating superior randomness compared to simple mapping.

### 2.2 Randomized DNA Base Sequence Design Rules for Data Storage

**Figure 2:**
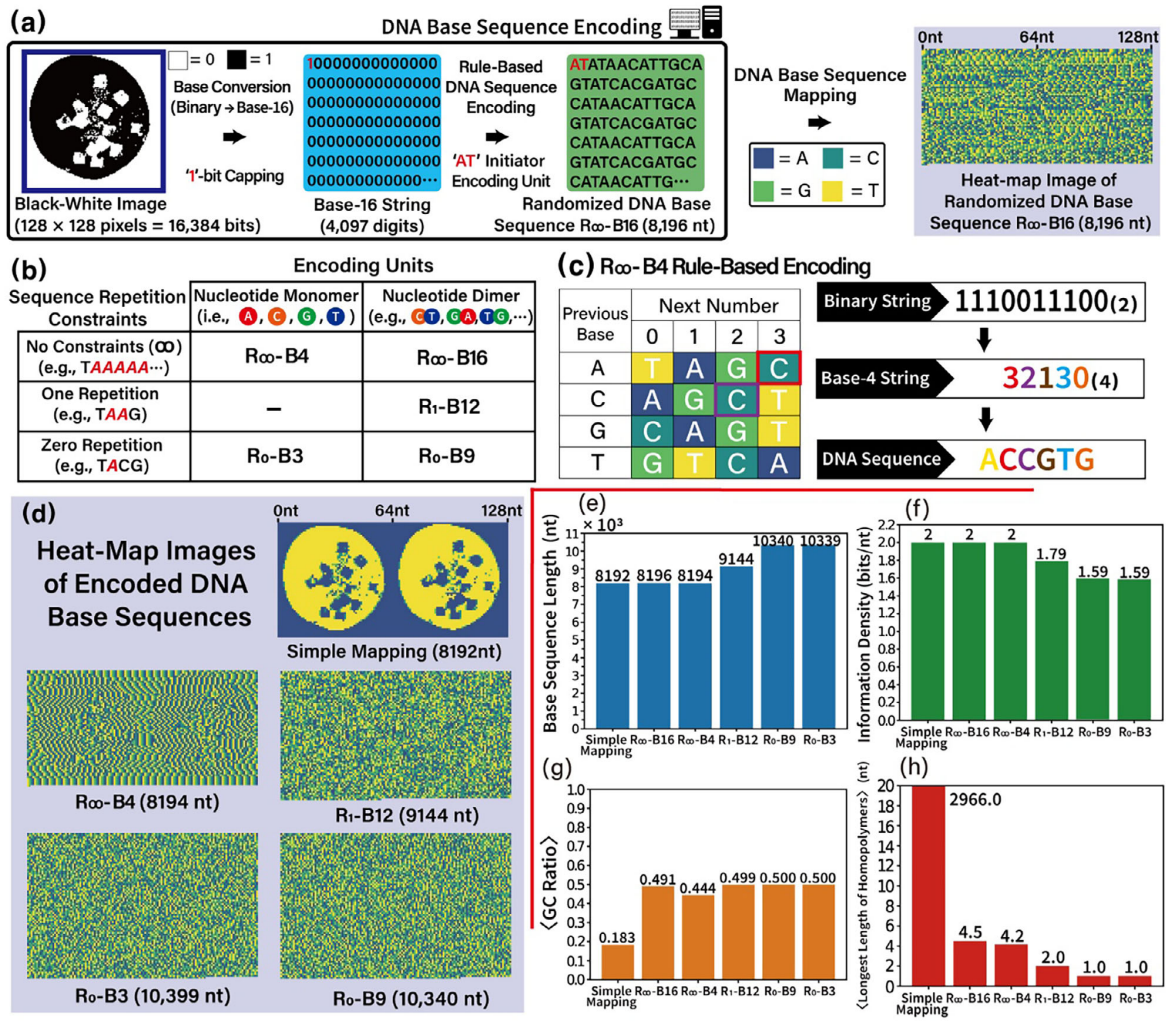
Properties of randomized DNA base sequence design rules and results of encoded DNA base sequences. a) The process of DNA base sequence encoding through randomized DNA base sequence design rule (e.g., R_∞_‐B16). The quality of sequence randomization is visually assessed using a heat‐map, where each of the four nucleotide bases (A, C, G, T) is represented by a distinct colour: blue for A, dark green for C, light green for G, and yellow for T. b) Characteristics of randomized DNA base sequence design rule R_N_‐B# (such as R_∞_‐B16, R_∞_‐B4, R_1_‐B12, R_0_‐B9, and R_0_‐B3). N and # in R_N_‐B# indicate the maximum allowable length of homopolymers and number of nucleotide‐based encoding units used, respectively. Here, R_∞_‐B4 and R_0_‐B3 employ monomer units for binary data encoding, while R_∞_‐B16, R_1_‐B12, and R_0_‐B9 utilize dimer units. c) Encoding the rule table of randomized DNA base sequence design rule R_∞_‐B4 and encoding an example conversion of a 10‐bit binary (1110011100_(2)_) to a DNA base sequence (ACCGTG). d) Visualization of DNA sequences encoded by different schemes (such as simple mapping, R_∞_‐B4, R_1_‐B12, R_0_‐B3, and R_0_‐B9) through heat‐map images. e–h) Analysis results of DNA sequences generated by six encoding schemes. These results include the total length of the sequences, information density, average GC ratio, and average longest homopolymer, derived from the six test images shown in Figure 1a. [Statistical analysis; data in (e–h) are presented in mean values. Data sample size is six (n = 6).]

**Figure** **2**a illustrates the workflow of randomized DNA base sequence encoding using R_∞_‐B16 (no limitation for homopolymer length in one encoded base sequence and another encoded sequence based on Base‐16 string converted from binary). The 16 384 (= 128 × 128)‐bit‐long original binary string needs to be converted into Base‐16 (hexadecimal) string to be encoded into randomized DNA base sequence through 16 (= 4^2^) nucleotide dimer encoding units (i.e., from AA, AC, AG, AT, to TA, TC, TG, TT). To prevent data loss of the leading “0”‐bit during base conversion from binary to hexadecimal, a “1”‐bit is added to the front of binary string before base conversion. The 4097‐digits of the Base‐16 string obtained from the 16385 binary bits is encoded into a randomized DNA base sequence via rule‐based DNA base sequence encoding R_∞_‐B16 (Table S2, Supporting Information), starting from an arbitrarily chosen initiator dimer encoding unit (e.g., AT). Consequently, an 8196‐nt‐long [= 2 (initiator) + 2 (dimer) × 4097] randomized DNA base sequence is obtained.

Figure 2b displays five randomized DNA base sequence design rules (i.e., R_∞_‐B16, R_∞_‐B4, R_1_‐B12, R_0_‐B9, and R_0_‐B3) used in this research. Those five encoding schemes can be classified into three groups (i.e., R_∞_‐B16/B4, R_1_‐B12, and R_0_‐B9/B3) based on sequence repetition constraints that control the maximum length of homopolymers by selectively allowing encoding units to be used in the following sequence. Five encoding schemes can also be classified into two groups based on the size of encoding unit (nucleotide monomer for R_∞_‐B4 and R_0_‐B3 and nucleotide dimer for R_∞_‐B16, R_1_‐B12, and R_0_‐B9).

Figure 2c shows an example of encoding a randomized DNA base sequence through rule‐based DNA base sequence encoding R_∞_‐B4. A 10‐bit binary string (1110011100_(2)_) needs to be converted into a Base‐4 string (32130_(4)_) to be encoded into a DNA base sequence via R_∞_‐B4. Using an arbitrarily selected nucleotide monomer encoding unit A (served as an initiator), the next encoding unit encoding 3 (red) in Base‐4 is determined as nucleotide base C (red box in encoding rule table R_∞_‐B4). The next nucleotide base encoding 2 (purple) in the Base‐4 string is determined as base C (purple box in encoding rule table R_∞_‐B4), as the previous encoding unit is base C. Consequently, DNA base sequence 5’‐ACCGTG‐3’ is obtained from binary string 1110011100_(2)_ through R_∞_‐B4 following the same procedure.

Figure 2d illustrates heat‐map images for DNA base sequences encoded by various encoding schemes. Four colours in the heat‐map image represent four nucleotides (i.e., blue for A, dark‐green for C, light‐green for G, and yellow for T). Although the lengths of encoded DNA base sequences slightly increase, the encoded DNA base sequences become relatively more randomized (as shown in heat‐map images) as the applied sequence repetition constraints become more stringent (simple mapping → R_∞_‐B16/B4 → R_1_‐B12 → R_0_‐B9/B3).

Figure 2e–h displays characteristic results of DNA base sequences encoded by six encoding schemes [without (in simple mapping) and with (in R_N_‐B#) randomization]. All six test images in Figure 1a are used for analysis. Figure 2e shows the lengths (per image) of DNA base sequences based on the six encoding schemes. The length of an encoded DNA base sequence slightly increases as the applied sequence repetition constraints become more stringent. Figure 2f illustrates the information densities of six encoding schemes. Information densities (bits/nt) are obtained by dividing the length of the original binary string by the length of the encoded DNA base sequence. Figure 2g shows the average GC ratios of DNA base sequences encoded through six encoding schemes. The average GC ratios become balanced when randomized DNA base sequence encoding rules are applied, while the average GC ratio of simple mapping shows a biased GC ratio. Figure 2h displays the average length of the longest homopolymer among encoded DNA base sequences for the six encoding schemes. The length of the longest homopolymer is effectively reduced in DNA base sequences encoded by randomized DNA base sequence design rules, whereas the longest homopolymer in simple mapping is extremely long. As we expected, due to applied sequence repetition constraints, the length of the longest homopolymer is 2 nt (1 nt) in a DNA base sequence encoded by R_1_‐B12 (R_0_‐B9/B3) (Table S3, Supporting Information).

A detailed quantitative comparison between the proposed randomized DNA base sequence design rules (RN–B#) and existing constraint‐based DNA coding algorithms (e.g., HEDGES, DNA Fountain, and yin–yang codec) is provided in Section S1 and Figures S1 and S2 (Supporting Information).

### 2.3 Characteristics of the Translational Active Particle Trajectory Model

**Figure 3:**
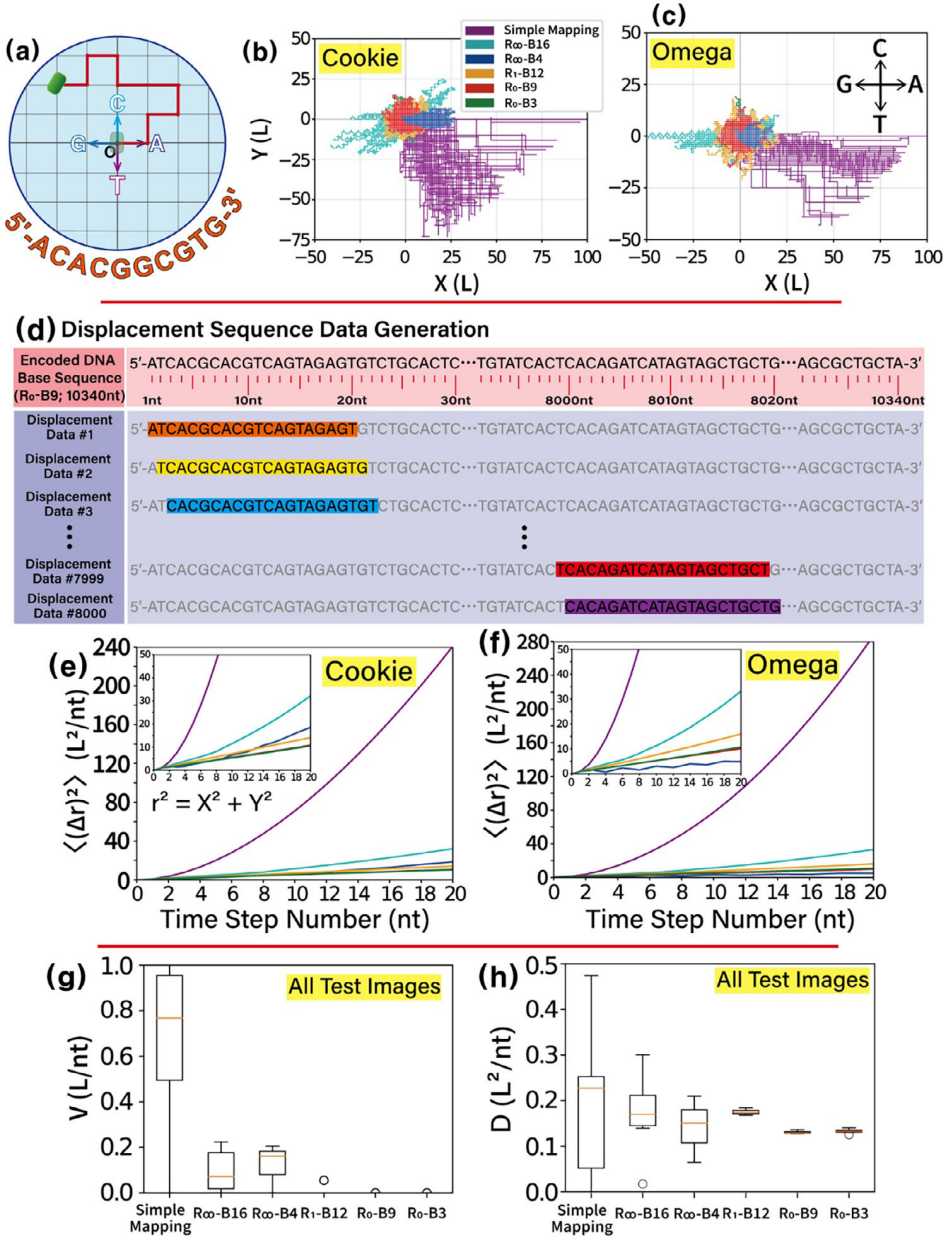
Characteristics and results of a 2D translational active particle trajectory model. a) Schematic illustration of particle trajectory. This panel illustrates the trajectory of a particle in 2D space, starting at the origin (0th‐step) and moving to its final position after 10 steps. The trajectory corresponds to a sample derived from a 10‐nt‐long DNA base sequence. b) Trajectories of particles in 2D space for DNA base sequences encoded by six encoding schemes (Cookie image). Particle trajectories in 2D space are shown for DNA base sequences encoded by six distinct encoding schemes: simple mapping, R_∞_‐B16, R_∞_‐B4, R_1_‐B12, R_0_‐B9, and R_0_‐B3. Each trajectory shows the initial position (0th‐step) and the final position after 100 steps, corresponding to a 100‐nt‐long DNA base sequence. A total of 80 particle trajectories is visualized for each encoding scheme, with the following assigned colours: purple (simple mapping), light blue (R_∞_‐B16), blue (R_∞_‐B4), orange (R_1_‐B12), red (R_0_‐B9), and green (R_0_‐B3). c) Trajectories of particles in 2D space for DNA base sequences encoded by six encoding schemes (Omega image). d) Schematic illustration of displacement sequence data generation from an encoded DNA base sequence. Each displacement sequence, consisting of 20 nt, overlaps with the neighbouring sequence by 19 nt. The displacement sequence data captures the distance (Δr(n)) between the final position (after n steps; n ≤ 20) and the initial position (origin). A total of 8000 displacement sequences per encoding scheme (e.g., R_0_‐B9) is analyzed to compute the mean square displacement (MSD, ⟨(Δr(n))^2^⟩) curve. e,f) Mean square displacement (MSD) analysis. MSD curves are computed for displacement sequences generated using the six encoding schemes, with results represented in distinct colours matching the encoding schemes in Figure 3b. Original binary data from the Cookie and Omega images are used as the basis for this analysis. g,h) Box diagrams of velocity (V) and diffusion coefficient (D) obtained using all six test images in Figure 1a. Box diagrams summarize the measured velocity and diffusion coefficient derived from the displacement sequences for the six encoding schemes. [Statistical analysis; data in (g,h) are presented as box plots showing the median, interquartile range (IQR = Q3 − Q1; 25th (Q1) to 75th (Q3) percentile as the box), and whiskers (extending to the smallest and largest values within Q1 – 1.5 × IQR and Q3 + 1.5 × IQR). Outliers are indicated with individual circles. Data sample size is six (n = 6).]

A 2D translational active particle trajectory model is used to evaluate the randomness of encoded DNA base sequences by measuring the mean square displacement (MSD) resulting from corresponding encoded DNA base sequences. In this model, each of the four nucleotide bases is represented as a unit vector in a distinct direction within a 2D plane: A = (L, 0), C = (0, L), G = (−L, 0), and T = (0, −L). **Figure** **3**a provides an example of active particle motion in a 2D space based on a 10‐nt DNA sequence. The particle moves a unit length L from the origin in the plane during each time step (corresponding to 1‐nt), guided by the sequence data (e.g., 5′‐ACACGGCGTG‐3′). When the DNA base sequence contains extended homopolymers (e.g., TAAAAACAAA), the particle motion becomes more biased (self‐propelled) in a particular direction, reflecting a lack of randomness. Conversely, a randomized base sequence (e.g., ACAGTGCTGA) results in an unbiased, random walk characteristic of Brownian motion. Thus, the model reveals that sequence patterns significantly influence particle motion, distinguishing between biased trajectories due to homopolymer stretches and random motion arising from a diverse, unstructured base sequence.

Figure 3b,c illustrates 2D particle trajectories derived from DNA base sequences encoded from the original Cookie [Omega] image using various encoding schemes, including simple mapping, R_∞_‐B16, R_∞_‐B4, R_1_‐B12, R_0_‐B9, and R_0_‐B3. DNA base sequences of up to 8000‐nt from the start of the sequence are analyzed for each encoding scheme. For each scheme, 80 trajectories, each consisting of 100‐nt time steps (= 8000 ÷ 100), are displayed within the 2D plane. The DNA base sequences encoded using simple mapping, characterized by multiple homopolymers, produce trajectories with larger displacements [Δr(n): the distance between the particle's final position (after n time steps) and the origin] and wider distributions compared to the other encoding schemes. This reflects the dominance of biased (self‐propelled) motion in simple mapping. Notably, the biased movements toward the fourth quadrant observed in the simple mapping trajectories suggest that the encoded sequences are predominantly composed of A and T nucleotides (resulting in a low GC ratio). In contrast, trajectories generated by R_∞_‐B16 and R_∞_‐B4 encoding schemes also show some degree of biased movement. However, the overall displacements of these trajectories are significantly reduced. This indicates that the DNA base sequences encoded by R_∞_‐B16 and R_∞_‐B4 are relatively more randomized compared to those from simple mapping. Meanwhile, the trajectories of DNA base sequences encoded by R_1_‐B12, R_0_‐B9, and R_0_‐B3 exhibit a circular distribution around the origin. This suggests that these encoding schemes impose effective restrictions on homopolymer length, resulting in minimal biased motion.

Figure 3d shows a schematic illustration of displacement sequence data generation. For a DNA base sequence encoded by each encoding scheme (e.g., R_0_‐B9), the 8020‐nt‐long sequences are used for randomness assessment. The length of a single displacement sequence obtained from the whole 8020‐nt‐long DNA base sequence is 20 nt. To obtain displacement sequence data from the given length of DNA base sequences (8020‐nt‐long), each 20‐nt‐long displacement sequence is obtained by 1‐nt parallel shifting (sliding) from previous displacement data, allowing 19‐nt bases to overlap with neighbouring displacement data. A total of 8000 displacement sequences per encoding scheme is analyzed to compute the mean square displacement (MSD, ⟨(Δr(n))^2^⟩) as a function of time step number.

Figure 3e,f display the mean square displacement curves for the Cookie and Omega images, respectively. The MSD for each time step (nucleotide) up to 20 nt long is calculated by averaging 8000 squared displacements from the origin at a given time step. Over short time intervals (Δn ≤ 20‐nt), the MSD (˂(Δr)^2^>) can be expressed as: ˂(Δr)^2^> = 4D·Δn + V^2^·Δn^2^, where D, Δn, and V represent the diffusion coefficient, time step number, and velocity (which corresponds to self‐propelled motion), respectively (see Sections S2 and S3 and Figure S3, Supporting Information). In DNA sequences encoded by simple mapping, which contain long homopolymer stretches, the MSD increases more rapidly in a parabolic manner, reflecting the presence of unmixed DNA sequences. In contrast, for DNA sequences generated with randomization principles, the MSD increase is significantly slower compared to sequences from simple mapping. The MSD of these randomized sequences follows a linear trend, proportional to the time step number, due to sequence repetition constraints that limit the length of homopolymers (e.g., 2 nt for R_1_‐B12 and 1 nt for R_0_‐B9/B3). Notably, the MSD of the R_∞_‐B4 sequence in the Omega image shows minimal increase, exhibiting a zig‐zag pattern, likely due to the periodic repetition of short sequence motifs (e.g., ACGTACGTACGT).

The characteristics of MSD (˂(Δr)^2^>) generated from DNA base sequences are analyzed using velocity (V) and the diffusion coefficient (D), which serve as parameters for sequence randomness. The value of V (measured in units of L/nt) is related to the lengths of homopolymers, while D (measured in L^2^/nt) quantifies sequence diffusion. Both parameters are derived by fitting MSD curves to the equation: ˂(Δr)^2^> = 4D·Δn + V^2^·Δn^2^. Figure 3e,f present box diagrams of the measured V and D, respectively, based on the six test images shown in Figure 1a. The diagrams show the median (orange line), interquartile range [IQR (= Q3 − Q1); 25th (Q1) to 75th (Q3) percentile as the box], and whiskers [extending to the smallest and largest values within (Q1 – 1.5 × IQR) and (Q3 + 1.5 × IQR)]. These diagrams allow quantitative comparisons across encoding schemes, highlighting differences in nucleotide positioning at each time step. As shown in Figure 3g, the V of simple mapping is the highest among the six encoding schemes due to the presence of long homopolymers in DNA base sequences encoded by simple mapping. Despite having the same information density (2 bits/nt), the velocities of R_∞_‐B16 and R_∞_‐B4 are significantly lower than those of simple mapping, as sequence randomization properties reduce homopolymer lengths. For R_1_‐B12, R_0_‐B9, and R_0_‐B3, the velocities are measured to be nearly zero, consistent with sequence repetition constraints that prevent homopolymers. The D values are shown in Figure 3h. In sequences encoded by R_∞_‐B16 and R_∞_‐B4, the reduced homopolymer lengths result in decreased D and lower V. Interestingly, DNA base sequences encoded by R_1_‐B12, R_0_‐B9, and R_0_‐B3 exhibit much smaller deviations in both V and D compared to those of simple mapping and R_∞_‐B16/B4. These findings highlight that the randomization principles applied in R_1_‐B12 and R_0_‐B9/B3 ensure higher quality for sequence randomness.

### 2.4 Characteristics of the Rotational Active Particle Trajectory Model

**Figure 4:**
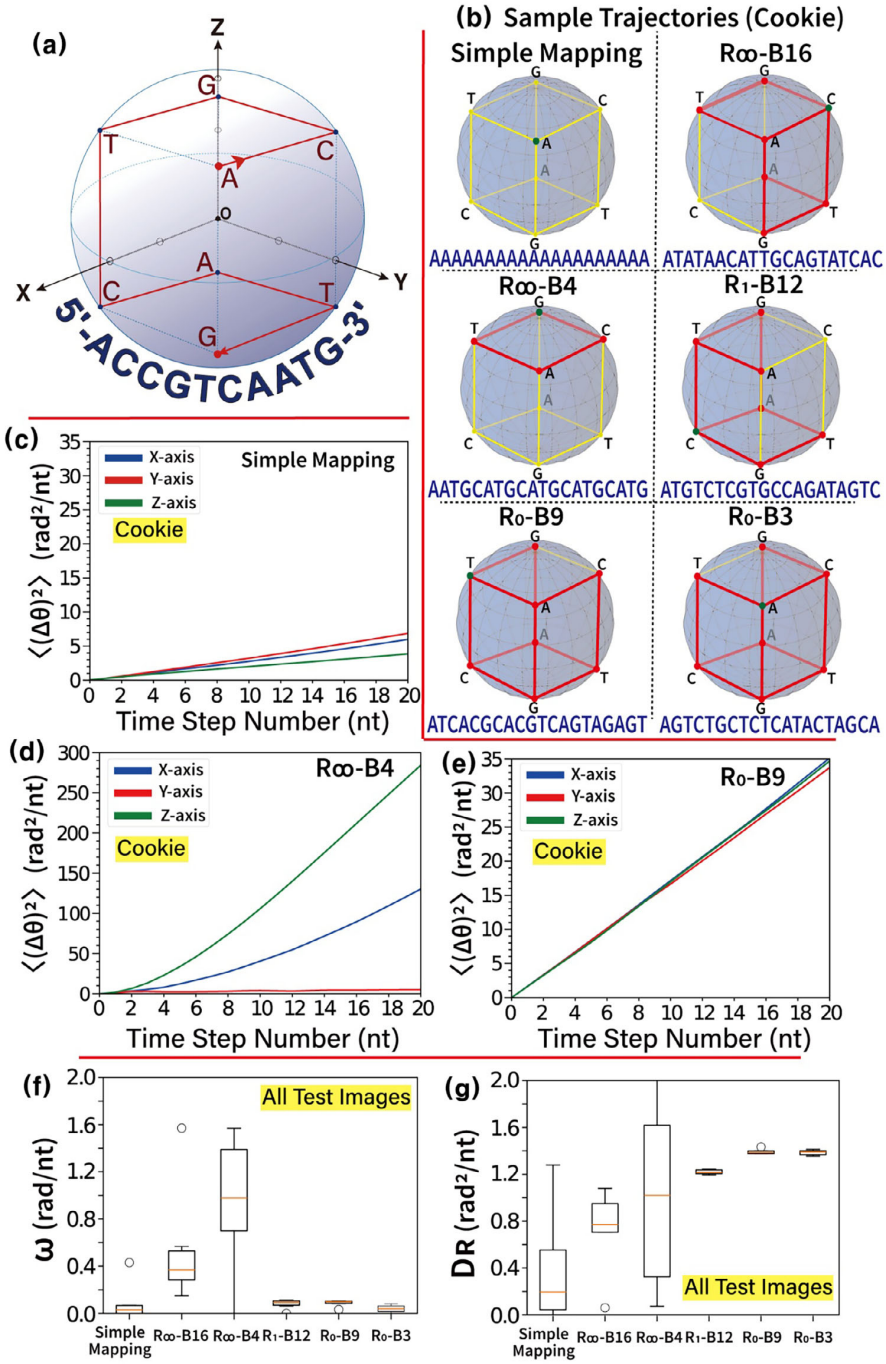
Characteristics and results of a 3D rotational active particle trajectory model. a) Schematic illustration of particle trajectory. This panel illustrates the trajectory of a particle in 3D space, starting at the corresponding base in the upper plane of a cube (0th‐step) and moving to its final position after ten steps. Upper and lower planes of a cube contain four bases in each corner for measuring angular characteristics of sequence trajectories. The trajectory corresponds to a sample derived from a 10‐nt‐long DNA base sequence. b) Sample trajectories of 20‐nt‐long DNA base sequences encoded by six encoding schemes (i.e., simple mapping, R_∞_‐B16, R_∞_‐B4, R_1_‐B12, R_0_‐B9, R_0_‐B3) (obtained by Cookie image) after 20‐nt time steps (final positions are marked with green dots). c–e) Mean square angular displacement (MSAD) analysis. MSAD curves are computed for displacement sequences generated using encoding schemes (Cookie image) such as simple mapping, R_∞_‐B4, and R_0_‐B9. MSAD curves on the x, y, and z axes are displayed in blue, red, and green curves, respectively. f,g) Box diagrams of angular velocity (ω) and rotational diffusion coefficient (D_R_) obtained using all six test images in Figure 1a. Box diagrams summarize the measured angular velocity and rotational diffusion coefficient derived from the displacement sequences for the six encoding schemes. [Statistical analysis; data in (f,g) are presented as box plots showing the median, interquartile range (IQR = Q3 − Q1; 25th (Q1) to 75th (Q3) percentile as the box), and whiskers (extending to the smallest and largest values within Q1 – 1.5 × IQR and Q3 + 1.5 × IQR). Outliers are indicated with individual circles. Data sample size is six (n = 6)].

Rotational active particle trajectory models evaluate sequence randomness of encoded DNA base sequences by corresponding DNA base sequences into displacement sequence data of an active particle in 3D space. In this space, four nucleotide bases are placed in opposing pairs at the eight vertices of a cube tangent to a sphere, as shown in **Figure** **4**a, to obtain angular displacement sequence data resulting from the movement of a particle per unit time step number (1 nt). A particle moves along the edge between two vertices where nucleotide bases are located per 1‐nt time step. Due to the movement at each time step, angular displacement (Δθ) at a perpendicular (π/2 radians) position occurs in two axes. For example, as shown in Figure 4a, the initial movement of nucleotide base A to C results in perpendicular angular displacement on the Y and Z axes. If the given DNA base sequence contains long homopolymers (e.g., GCTTTTTTAG), rotational mobility of the particle decreases as the particle remains in a fixed position during consecutive sequencing of base T. Every permutation of four nucleotide bases (i.e., ACGT, ACTG, AGCT, AGTC, ATCG, and ATGC) can be obtained by particle rotation along the edges of each face of the cube. The outward‐directed curl of each face of the cube corresponds to one of six permutations of the four nucleotide bases (e.g., permutations ACGT, ATGC, ATCG correspond to +Z‐, −Z‐, +X‐directions in Figure 4a, respectively). Therefore, the sequence of four nucleotide bases with periodic repetition (e.g., ACGTACGT) corresponds to rotational motion in a single face in 3D space, resulting in highly angularly biased (self‐propelled) motion of the active particle in a single direction. For a randomized DNA base sequence (e.g., ACAGTGCTGA), however, the randomized displacement moves the particle along multiple paths in 3D space, resulting in an angular random walk along each axis.

Figure 4b illustrates the trajectories in 3D space for the first 20‐nt base sequences of DNA base sequences encoded using six encoding schemes: simple mapping, R_∞_‐B16, R_∞_‐B4, R_1_‐B12, R_0_‐B9, and R_0_‐B3. The original binary data for encoding are represented by the Cookie image displayed in Figure 1a. The trajectory of the DNA base sequence encoded by simple mapping demonstrates stationary motion, resulting from a homopolymer composed entirely of nucleotide base A. In contrast, the trajectory of the sequence encoded by R_∞_‐B4 exhibits rotational motion along the Z‐axis, driven by the ATGC‐repeat‐encoded DNA base sequence. As the encoding schemes become more randomized (progressing from R_∞_‐B4 to R_1_‐B12 to R_0_‐B9/B3), the total path length of the particle trajectory in 3D space increases over the same time interval (20 nt, as shown in Figure 4b). This reflects a transition in particle motion from simple rotational movement to a random walk in 3D space.

Figure 4c–e presents a mean square angular displacement (MSAD) analysis computed from displacement sequences generated using various encoding schemes (Cookie image). Using the same methods as applied in the translational active particle trajectory model, a total of 8000 displacement sequences, each 20 nt in length, is obtained from DNA base sequences encoded by each scheme. The MSAD, defined as ˂(Δθ)^2^> = 2D_R_·Δn + ω^2^·Δn^2^ (where D_R_ is the rotational diffusion coefficient and ω is the angular velocity), is calculated along the three directions (X, Y, and Z axes) at each time step. The calculation involved averaging 8000 square angular displacements from the origin in each direction based on the particle's positional changes in 3D space at each time step. For the simple mapping scheme, as shown in the trajectory in Figure 4b, the DNA base sequence primarily contains long homopolymers. Consequently, the MSAD curves for simple mapping (Figure 4c) exhibit relatively small linear increases over the 20‐nt time interval, indicating low angular mobility of the particle in 3D space. In contrast, the MSAD curves for R_∞_‐B4 demonstrate a rapid parabolic increase, particularly in the Y and Z directions (prominently in the ±Z direction). This behaviour is attributed to the periodic repetition of the encoded DNA base sequence (e.g., ATGC‐repeat sequences, primarily corresponding to motion in the −Z direction). Meanwhile, the MSAD curves for R_0_‐B9 exhibit a linear increase across all directions (X, Y, and Z), consistent with the properties of diffusive particles. This linearity arises from the particle's random walk motion in 3D space, driven by the randomized DNA base sequence generated via R_0_‐B9.

Figure 4f,g show box diagrams of measured angular velocity ω and rotational diffusion coefficient D_R_ to evaluate the encoded DNA base sequence randomness through rotational active particle trajectory model in 3D space. The six test images in Figure 1a are used as original binary data. Angular velocity ω and rotational diffusion coefficient D_R_ (by calculating the vector magnitude of the three components of ω and D_R_ in X, Y, and Z directions [i.e., ω = (ω_X_^2^ + ω_Y_^2^ + ω_Z_^2^)^0.5^ and D_R_ = (D_RX_^2^ + D_RY_^2^ + D_RZ_^2^)^0.5^]) are obtained through MSAD curves.

In the translational active particle trajectory model (Figure 3), the length of homopolymers in encoded DNA base sequences significantly influences unbiased motion, resulting in relatively higher V and D for simple mapping, while lower V and D are observed for R_∞_‐B4. Conversely, in the rotational active particle trajectory model, periodic repetitions in base sequences (e.g., AGTCAGTCAGTC) primarily affect particle motion in 3D space. This leads to relatively higher ω and D_R_ in R_∞_‐B4, while lower ω and D_R_ are observed in simple mapping. For R_1_‐B12 and R_0_‐B9/B3, ω is measured to be near zero, as indicated by the linearity of the obtained MSAD curves. This behaviour results from sequence repetition constraints that limit the length of homopolymers in encoded DNA base sequences (e.g., 2 nt for R_1_‐B12 and non‐homopolymer sequences for R_0_‐B9/B3). Additionally, R_1_‐B12 exhibits a lower D_R_ compared to R_0_‐B9 and R_0_‐B3, as the particle motion for DNA base sequences encoded by R_1_‐B12 is more constrained, leading to reduced mobility compared to R_0_‐B9/B3. Similar to the results in the translational active particle trajectory model, ω and D_R_ for simple mapping and R_∞_‐B16/B4 show broad distributions due to weak sequence randomization capabilities. In contrast, ω and D_R_ for R_1_‐B12 and R_0_‐B9/B3 converge to nearly constant values with minimal deviations, reflecting the strong sequence randomization capabilities imposed by their sequence repetition constraints.

### 2.5 Inverse Ising Model

**Figure 5:**
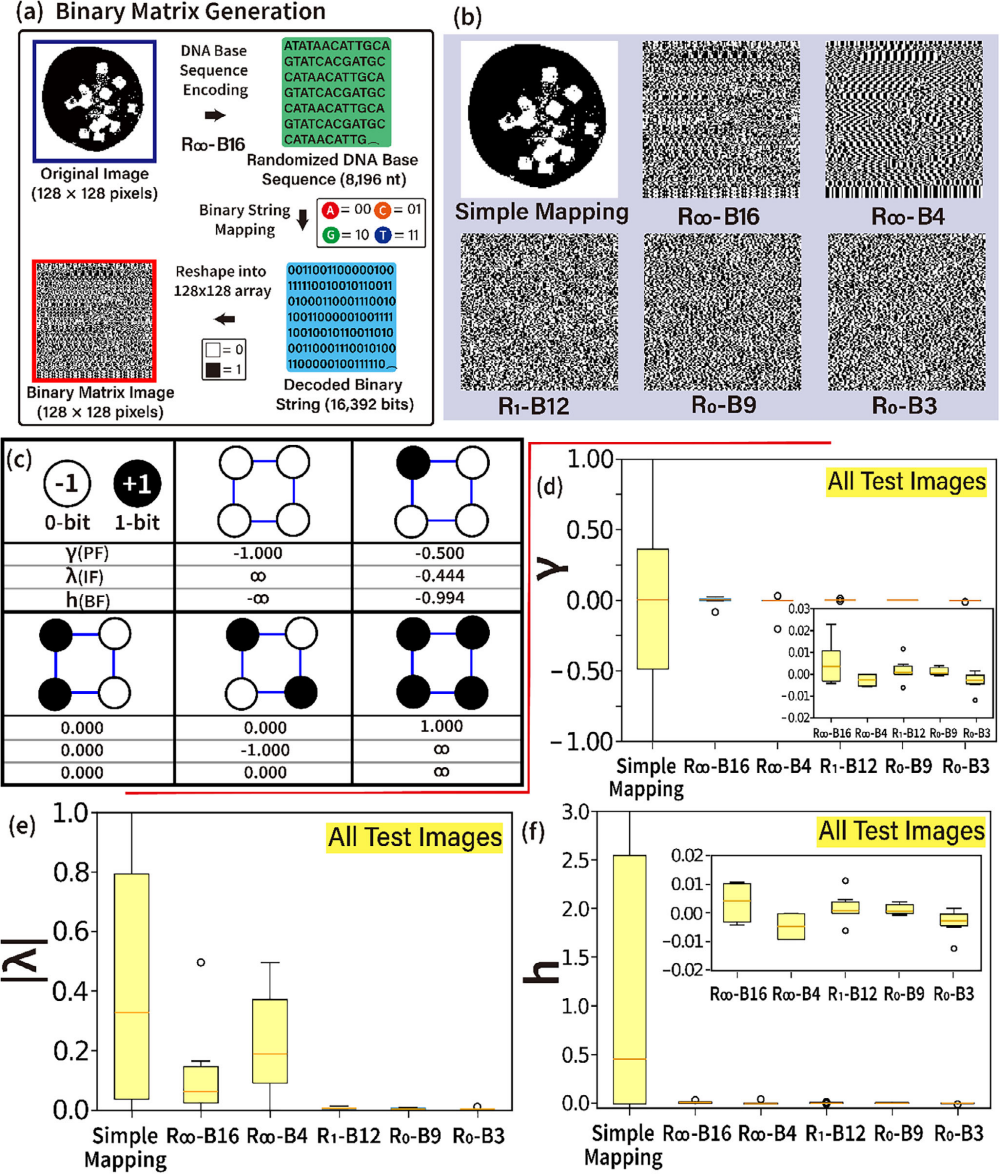
Randomization assessment of encoded DNA base sequences via the inverse Ising model. a) Workflow for binary matrix generation. A decoded binary string is created by mapping nucleotide bases (A → 00, C → 01, G → 10, T → 11) into binary form. These nucleotide bases originate from sources such as the Cookie image, following a specific design rule (e.g., R_∞_‐B16). The decoded binary sequence is then structured into a 128 × 128 binary matrix, where 16 384 (= 128 × 128) bits are extracted from the total 16392‐bit decoded sequence. b) Binary matrices from different encoding schemes. The six encoding schemes, simple mapping, R_∞_‐B16, R_∞_‐B4, R_1_‐B12, R_0_‐B9, and R_0_‐B3, are used to generate binary matrices from a sample test image (e.g., the Cookie image). Each encoding method produces distinct binary matrix images, revealing differences in data organization and randomness. c) Implementation of binary matrix assemblies to the inverse Ising model. Binary matrix units (tetramer representation) are analyzed using the inverse Ising model. Each unit consists of two nodes representing binary states (white for 0‐bit, black for 1‐bit), corresponding to spin‐up and spin‐down states. Five possible unit assemblies are illustrated, along with their associated inverse Ising model parameters: polarization factor (PF, γ), interaction factor (IF, λ), and bias factor (BF, *h*). d–f) Quantitative analysis of encoding schemes. The parameters γ, |λ|, and *h* are quantitatively compared across the six encoding schemes, based on binary matrices derived from all test images shown in Figure 1a. These comparisons provide insights into the randomness, interactions, and bias characteristics of each encoding approach. [Statistical analysis; data in (d–f) are presented as box plots showing the median, interquartile range (IQR = Q3 − Q1; 25th (Q1) to 75th (Q3) percentile as the box), and whiskers (extending to the smallest and largest values within Q1 – 1.5 × IQR and Q3 + 1.5 × IQR). Outliers are indicated with individual circles. Data sample size is six (n = 6)].

We introduce the inverse Ising model as another method for randomization assessment of encoded DNA base sequences quantitatively. **Figure** **5**a,b illustrate the process of binary matrix generation and representative binary matrices obtained from randomized DNA base sequences. To generate randomized binary matrices based on encoded DNA sequences (e.g., using randomized encoding rules such as R_∞_‐B16), an 8196‐nt‐long DNA sequence is first converted into a 16 392‐bit binary string (= 8196 × 2) through simple mapping. The binary string is then arranged into a 128 × 128 binary matrix, where the first 16384 bits are mapped sequentially. Figure 5b presents representative binary matrices generated using six encoding schemes, with the Cookie image as an example. When simple mapping is applied, the resulting binary matrix retains the original Cookie image since the encoding and decoding schemes remain identical. However, as encoding schemes impose stricter sequence repetition constraints (progressing from R_∞_‐B16/B4 → R_1_‐B12 → R_0_‐B9/B3), the binary matrices exhibit an increasingly randomized distribution of black and white cells. This indicates a higher degree of sequence randomization.

The inverse Ising model, a fundamental framework in statistical physics, is employed to quantitatively assess sequence randomness using characteristic parameters: polarization factor (PF, γ), interaction factor (IF, λ), and bias factor (BF, *h*). Figure 5c illustrates the implementation of binary matrix assemblies within the inverse Ising model. Binary matrix units, represented as tetramers, are analyzed using this model. Each unit contains two nodes representing binary states—white for 0‐bit (spin‐down, −1) and black for 1‐bit (spin‐up, +1). Five possible unit assemblies are depicted, along with their corresponding inverse Ising parameters: γ, λ, and *h*. Polarization factor (PF, γ) measures the density of the majority node in a binary matrix, with values ranging from −1 to 1 (i.e., −1 ≤ γ ≤ 1). The interaction factor (IF, λ) quantifies the interaction strength between neighbouring nodes, ranging from −1 to ∞ (−1 ≤ λ ≤ ∞). The bias factor (BF, *h*) reflects the intrinsic properties of a binary matrix, accounting for the unequal binding probabilities of the two available node states, with values from −∞ to ∞ (−∞ ≤ *h* ≤ ∞).

Consider an N × M binary matrix configuration, such as $\left[\begin{array}{cc} 1 & 0\\ 0 & 0 \end{array}\right]$, where N = M = 2. The spin‐state of each node in this configuration can be represented as $\left[\begin{array}{cc} \sigma_{1} & \sigma_{2}\\ \sigma_{3} & \sigma_{4} \end{array}\right]=\left[\begin{array}{cc} +1 & -1\\ -1 & -1 \end{array}\right]$, where σ_i_ denotes the spin value of the i^th^ node. The total number of nodes in the system is given by N_T_ = N × M = 2 × 2 = 4, while the total number of node connections is calculated as N_n_ = (N − 1) × M + (M − 1) × N = 4. Additionally, the total number of touching node connections is determined as N_t_ = 12 × (N − 1) × (M − 1) – 4 = 8. To compute the inverse Ising parameters, two sums are essential. The first is the summation over all spin states, given by Σ_i_ σ_i_ = (−1) × N_0_ + (+1) × N_1_, where N_0_ and N_1_ represent the number of 0‐bits and 1‐bits, respectively. In the given matrix, this results in Σ_i_ σ_i_ = (−1) × 3 + (+1) × 1 = −2. The second key summation accounts for pairwise spin interactions among connected (i.e., nearest neighbors) nodes, expressed as Σ_i,j_ σ_i_σ_j_ = σ_1_σ_2_ + σ_2_σ_4_ + σ_4_σ_3_ + σ_3_σ_1_. Substituting the given spin values, (−1) + (+1) + (+1) + (−1) = 0. With these parameters, the polarization factor, defined as γ(σ_i_, N_T_) = (Σ_i_ σ_i_) / N_T_, is calculated as γ = (−2)/4 = −0.5. Similarly, the interaction factor λ(σ_i_ σ_j_, N_T_, N_n_, N_t_) and the bias factor *h*(σ_i_ σ_j_, N_T_, N_n_, N_t_) are computed, yielding values of 0.444 (i.e., 9/16) and −0.994, respectively. These values provide insight into the system's spin interactions and overall behavior within the Ising model framework. Further details on the parameter formulas are provided in the Section S4 (Supporting Information).

Figure 5d–f present a numerical analysis of the polarization factor γ, interaction factor λ, and bias factor *h* applied to various test images in different sequence designs. These analyses demonstrate the model ability to recognize variations in binary matrices as the sequence design changes. The polarization factor γ, shown in Figure 5d, indicates which bit dominates the binary matrix for a given sequence design. If most of the bits in the binary matrix are 1s, γ takes a positive value; otherwise, it is negative. More highly randomized sequences result in values close to zero, with only minor deviations. In contrast, less randomized sequences produce relatively larger deviations, indicating an unmixed sequence structure. The absolute value of the interaction factor, ∣λ∣, as illustrated in Figure 5e, provides a comparative measure of sequence randomization across randomized sequence designs, including simple mapping, R_∞_‐B16, R_∞_‐B4, R_1_‐B12, R_0_‐B9, and R_0_‐B3. This factor characterizes interactions between neighbouring nodes, revealing the degree of mixing or clustering within the binary matrix. Higher values of ∣λ∣ indicate clustering, where binary values form contiguous or structured groupings, whereas lower values of ∣λ∣ suggest a more random distribution. Notably, the trends observed in R_1_‐B12, R_0_‐B9, and R_0_‐B3 exhibit very small ∣λ∣ values, indicating that these designs effectively produce well‐mixed binary matrix data, corresponding to highly randomized DNA sequences. The bias factor *h*, presented in Figure 5f, exhibits a behavior similar to that of the polarization factor γ. This is expected, as *h* is an increasing function of γ, meaning that its behavior closely follows the variations of γ. By comparing γ, λ, and *h*, the inverse Ising model effectively reveals the bit composition and interaction structure of binary matrix data across sequence designs.

### 2.6 3‐Input 1‐Output Logic Algorithm System

**Figure 6:**
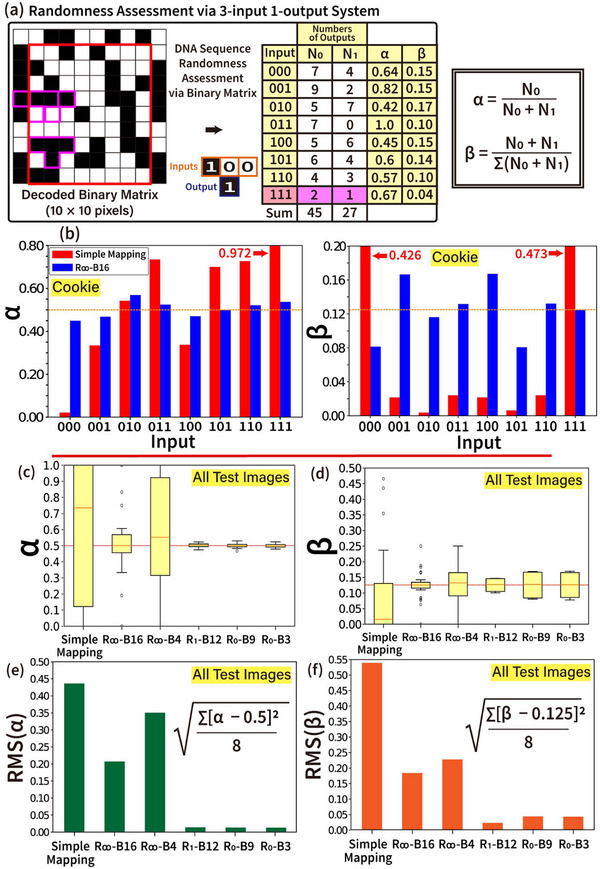
Randomness assessment using the 3‐input 1‐output logic algorithm model. a) The assignment of randomness assessment parameters, such as the ratio of 0‐bit outputs per number of bits at a given input [i.e., α(input = i_1_i_2_i_3_)] and the ratio of number of bits at a given input per total number of bits [i.e., β(i_1_i_2_i_3_)]. Parameters and numbers are obtained using a measurable binary matrix (marked with a red box). b) Results of measured α and β with respect to each 3‐bit input (i.e., 000, 001, 010, 011, 100, 101, 110, 111) from randomized binary matrices (red from simple mapping and blue from R_∞_‐B16). Expectation values of α (= 0.5) and β (= 0.125) for a perfectly randomized binary matrix are displayed with orange dotted lines. c,d) Box diagrams of distributions of measured α and β for eight available inputs (obtained using all six test images shown in Figure 1a). The diagrams display the median (orange line), interquartile range (25–75% shown as the box), and the whiskers (minimum to maximum range within 1.5 times the interquartile range). Outliers are indicated with individual circles. e,f) Root mean square (RMS) of α and β obtained from distributions of measured α and β. RMS(α) and RMS(β) quantitatively provide the degree of randomness of randomized DNA base sequences. [Statistical Analysis; Data in (c,d) are presented as box plots showing the median, interquartile range (IQR = Q3 − Q1; 25th (Q1) to 75th (Q3) percentile as the box), and whiskers (extending to the smallest and largest values within Q1 – 1.5 × IQR and Q3 + 1.5 × IQR). Outliers are indicated with individual circles. Data sample size in (c–f) is six (n = 6).]

The randomness assessment method using a 3‐input 1‐output logic algorithm system is illustrated in **Figure** **6**a. This system consists of eight possible 3‐input combinations (000, 001, 010, 011, 100, 101, 110, 111) and two possible 1‐output values (0 or 1). To evaluate randomness, two key parameters are introduced: α(i_1_i_2_i_3_), which represents the ratio of 0‐bits in the output to the total number of output bits for a given input, and β(i_1_i_2_i_3_), which denotes the proportion of occurrences of a specific input among all input occurrences. These are defined as α(i_1_i_2_i_3_) = N_0_ / (N_0_ + N_1_), where N_0_ and N_1_ represent the numbers of 0‐bits and 1‐bits at a given input, respectively, and β(i_1_i_2_i_3_) = (N_0_ + N_1_) / Σ(N_0_ + N_1_), where (N_0_ + N_1_) is the number of bits corresponding to a specific input, and Σ(N_0_ + N_1_) is the total number of bits across all inputs.

These parameters can be driven from a sample 10 × 10‐pixel‐size decoded binary matrix, as shown in Figure 6a, using a measurable binary matrix (marked with a red box) within the decoded binary matrix. For example, with (111) input, α(111) is calculated as 0.667 (i.e., 2 / 3) since two of the three output bits corresponding to the (111)‐input (marked with pink lines) are 0. Similarly, β(111) is measured as 0.042 (i.e., 3 / 72), as there are three occurrences of the (111)‐input among the total bits in the measurable binary matrix. The total number of bits in the measurable binary matrix is determined as (n − 1) × (n − 2), which in this example equals 72 (i.e., 9 × 8). This is also equal to Σ(N_0_ + N_1_) = ΣN_0_(i_1_i_2_i_3_) + ΣN_1_(i_1_i_2_i_3_) = 45 + 27 = 72. These calculations provide a structured approach to assessing randomness based on the binary matrix representation of inputs and outputs.

Figure 6b presents the measured values of α and β for each of the eight possible 3‐input combinations (000, 001, 010, 011, 100, 101, 110, 111) derived from two randomized binary matrices. The red bars represent results from simple mapping, while the blue bars correspond to the R_∞_‐B16 method. Decoded binary matrices are constructed by rearranging the binary sequences originally obtained from simple mapping and randomized DNA base sequences encoding Cookie image data into a 128 × 128‐pixel size, as discussed in Figure 5a. The expected values for a perfectly randomized binary matrix are indicated by the orange dotted lines, with α at 0.5 (= 1/2, since each bit has two possible values) and β at 0.125 (= 1/8, as there are eight possible 3‐input combinations). In the binary matrix generated through simple mapping, α and β values show noticeable bias, reflecting the lack of randomness in the corresponding DNA base sequences. Conversely, the binary matrix obtained using the R_∞_‐B16 method demonstrates greater balance between α and β values (compared to values from simple mapping), highlighting the effectiveness of the R_∞_‐B16 design rule in producing well‐randomized DNA sequences.

Figure 6c,d present box plots of the measured parameters α and β across six encoding schemes, using all test images from Figure 1a. These diagrams illustrate the median (orange line), interquartile range (25–75% as the box), and whiskers (minimum to maximum range within 1.5 times the interquartile range). In general, as the sequence randomization constraints in the encoding scheme become more stringent (i.e., R_1_‐B12, R_0_‐B9, and R_0_‐B3 compared to simple mapping, R_∞_‐B16, and R_∞_‐B4), the deviations of α and β narrow, with mean values closer to the expected levels, indicating improved balance in encoded DNA base sequences. To quantify the randomness of encoded DNA base sequences, the root mean square (RMS) of parameters α and β is evaluated across six encoding schemes, as shown in Figure 6e,f. As expected, higher RMS(α) and RMS(β) values are observed in encoding schemes such as simple mapping, R_∞_‐B16, and R_∞_‐B4, indicating more bias in the α and β distributions. In contrast, encoding schemes with more stringent sequence repetition constraints, such as R_1_‐B12, R_0_‐B9, and R_0_‐B3, exhibited lower RMS(α) and RMS(β) values. This reflects a more balanced randomness, as these schemes strictly control homopolymer lengths in the encoded DNA base sequences.

### 2.7 PCR Amplification and Sanger Sequencing

**Figure 7:**
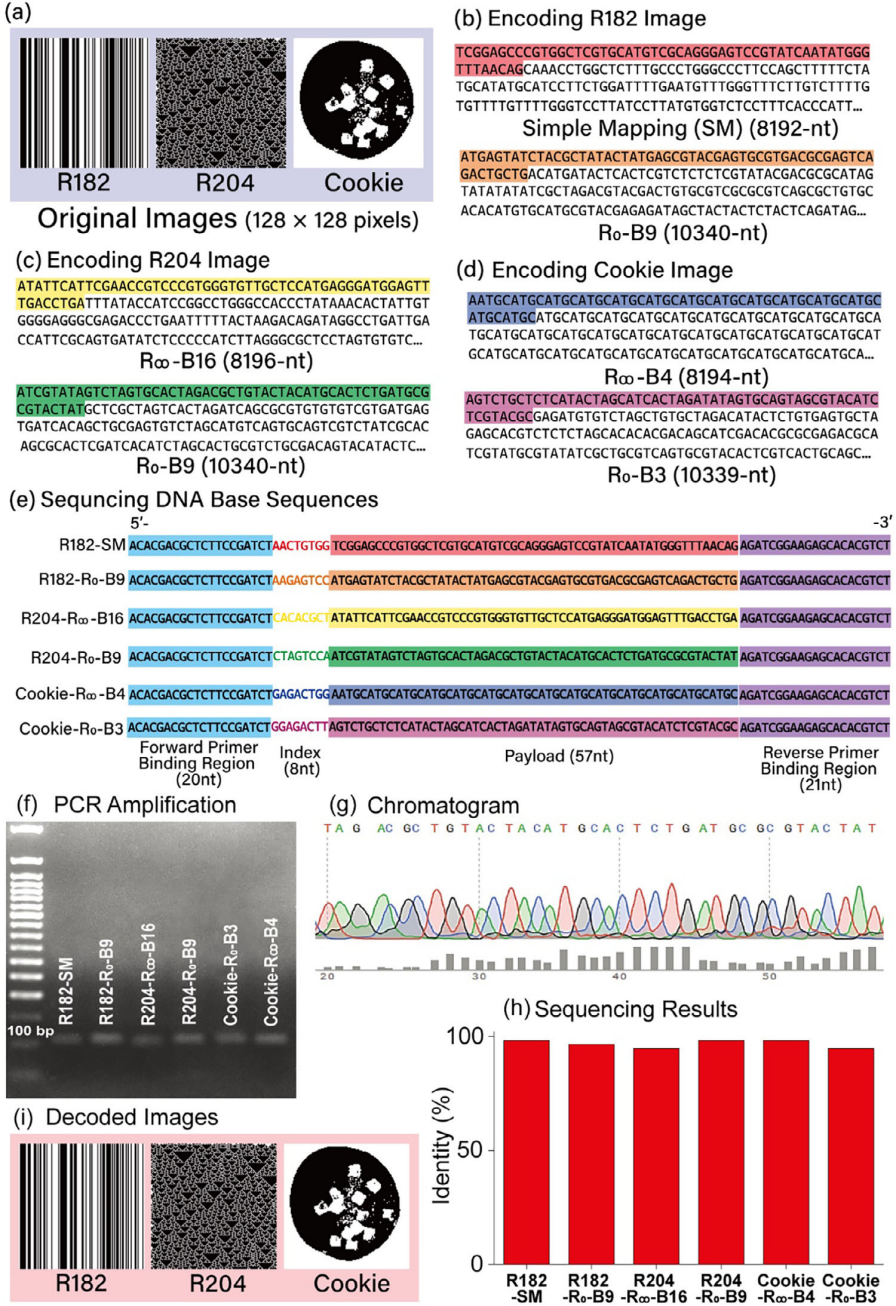
Experimental workflow of DNA sequencing for obtaining decoded images. a) Three original test images (128 × 128 pixels, black‐white binary) used for DNA sequencing. b–d) DNA base sequences encoded using simple mapping and randomized DNA base sequence rules. The initial 57‐nt‐long sequences (highlighted regions) within the entire encoded DNA sequences serve as the payload for sequencing. e) DNA strand preparation for PCR amplification and Sanger sequencing. Each 106‐nt‐long strand consists of forward and backward primer binding regions (20‐nt and 21‐nt, respectively), an index region (8‐nt), and a payload region (57‐nt). f) 2% agarose gel image showing the results of PCR amplification. g) Representative chromatogram of the payload region from R204‐R_0_‐B9. h) Sequencing identity of individual DNA strands. i) Final decoded images reconstructed from the read DNA base sequences. [Statistical analysis; data sample size in (h) is one (n = 1).]

The sequencing workflow for DNA base sequences encoded by different schemes is illustrated in **Figure** **7** to experimentally validate the feasibility of sequencing DNA base sequences (obtained by randomized DNA base sequence design rules such as simple mapping, R_∞_‐B16, R_∞_‐B4, R_0_‐B9, and R_0_‐B3) containing original image data. The three 128 × 128 pixel black‐white images (R182, R204, and Cookie) shown in Figure 7a serve as the original binary data. For the R182 image, DNA base sequences of 8192‐nt and 10 340‐nt are generated through simple mapping and R_0_‐B9 encoding, respectively (Figure 7b). The R204 image is encoded using R_∞_‐B16 and R_0_‐B9, resulting in DNA base sequences of 8196‐nt (R_∞_‐B16) and 10340‐nt (R_0_‐B9) (Figure 7c). The Cookie image is encoded using R_∞_‐B4 and R_0_‐B3, producing DNA base sequences of 8194‐nt (R_∞_‐B4) and 10339‐nt (R_0_‐B3) in length (Figure 7d).

To enhance handling and improve sequencing efficiency, the DNA base sequences generated through simple mapping and randomized DNA base sequence design rules were segmented into shorter fragments, each 57‐nt in length. The initial 57‐nt sequences, highlighted within the complete encoded DNA sequences, served as the payload for DNA base sequencing.

Each DNA strand, measuring 106‐nt in total, included forward and backward primer binding regions, an index region, and a payload region. These structured strands were utilized for downstream PCR amplification and Sanger sequencing, as illustrated in Figure 7e.

Each DNA strand underwent PCR amplification, followed by quality control verification using a 2% agarose gel to confirm the size of the amplified products (Figure 7f). After amplification and purification, Sanger sequencing was performed on each strand. A representative chromatogram for the payload region of the R204‐R_0_‐B9 sequence is shown in Figure 7g, where each base call is accompanied by a Phred25 quality score, represented by grey bars below the chromatogram. To evaluate sequencing accuracy, the sequence identity percentage, defined as (number of errors in payload / length of the payload) × 100, was analyzed (Figure 7h). The sequence identities for all five encoding schemes range between 95% and 98%. Ultimately, the decoded images were successfully reconstructed from the read DNA base sequences, as shown in Figure 7i.

## 3 Conclusion

We demonstrated that physics‐based quantitative assessment provides an effective means to optimize DNA sequence design for digital data storage. Encoding schemes incorporating strict homopolymer constraints (e.g., R_1_‐B12, R_0_‐B9, R_0_‐B3) consistently yielded highly randomized sequences with minimal compositional bias, as confirmed by active particle trajectory dynamics, Ising spin interaction metrics, and input–output distribution analysis. These improvements translated into experimentally verified high decoding accuracy, underscoring the practical feasibility of the approach. Our framework offers a generalizable methodology for evaluating and engineering DNA sequences, bridging theoretical modeling and experimental validation. By integrating physical modeling with sequence engineering, this strategy advances the development of robust molecular data storage systems capable of long‐term, high‐density information preservation. Although images were used as representative digital inputs in this study, the proposed framework is inherently data‐agnostic. Since all data types—including text, audio, and video—are first converted to binary strings prior to DNA sequence encoding, the same randomization rules and evaluation metrics are equally applicable. Thus, the physics‐based assessment framework can be broadly extended to optimize DNA sequence design for diverse information types, ensuring reliable molecular storage beyond image datasets.

## 4 Experimental Section

### DNA Sample Preparation

Each DNA sequence, provided as a text file, contained strand indices and forward/reverse primer‐binding regions to enable efficient PCR amplification. The sequence structure was: 5′‐ACA CGA CGC TCT TCC GAT CT (20 nt) – strand index (8 nt) – payload sequence (57 nt) – AGA TCG GAA GAG CAC ACG TCT (21 nt)‐3′. Oligonucleotides were synthesized by Integrated DNA Technologies (IA, USA) using a desalting purification method (Figure 7).

### PCR Amplification

PCR was performed on a TaKaRa PCR Thermal Cycler Dice Touch (Korea) with an initial DNA concentration of 5 ng/µL. Each 25 µL reaction mixture contained 1.0 µL DNA sample, 12.5 µL Q5 PCR Master Mix, 9.0 µL mQ water, and 1.25 µL each of 10 µm forward and reverse primers. Cycling conditions were i) initial denaturation: 98 °C, 1 min, ii) 20 cycles of 98 °C, 10 s → 69 °C, 30 s → 72 °C, 30 s; and iii) final extension: 72 °C, 3 min. Amplification products were analyzed via 2% agarose gel electrophoresis using a 50 bp DNA ladder (GenDEPOT iVDYE) as a reference. Product concentration was quantified with a NanoDrop spectrophotometer (Thermo Fisher Scientific) to verify yield and purity (Figure 7).

### PCR Product Sequencing

Purified PCR products were prepared for Sanger sequencing (Cosmo Genetech, Korea) using 50 ng µL^−1^ of product with 5 pmol µL^−1^ of both forward and reverse primers. Sequencing generated FASTQ files and chromatograms for each sample (Figure 7).

### Statistical Analysis

Statistical analysis in this study is conducted using Python (Matplotlib version 3.9.2). Quantitative results are presented as mean values or box plots indicating median and interquartile range (IQR = Q3 – Q1), as specified in the figure legends. The sample size for each dataset is provided in the corresponding figure captions.

## Conflict of Interest

The authors declare no conflict of interest.

## Author Contributions

S.S., T.H.N.V., and S.P. designed the research. S.S., T.H.N.V., and S.P. analyzed the data. A.T. and T.B.N.N. performed the experiments and collected the data. S.S., T.H.N.V., S.P., A.T., and T.B.N.N. performed the investigation. S.S., T.H.N.V., and S.P. drafted the original manuscript. S.K. and S.H.P. conceived and supervised the study and revised the manuscript. All authors read and approved the final manuscript.

## Supporting information



Supporting Information

## Data Availability

The data that support the findings of this study are available in the supplementary material of this article.
