# Supplementary material for: Quantitative Assessment of Randomized DNA Base Sequences Using Multi‐Model Physical Analysis for High‐Fidelity Data Storage
Source: Adv Sci (Weinh). 2025 Nov 21;13(7):e17208. doi: 10.1002/advs.202517208 (PMC12866812; doi:10.1002/advs.202517208)
Supplement: Supplementary file 1 — Supporting Information [file ADVS-13-e17208-s001.docx]

Supporting Information

**Quantitative Assessment of Randomized DNA Base Sequences Using Multi-Model Physical Analysis for High-Fidelity Data Storage**

*Seongjun Seo, Thi Hong Nhung Vu, Anshula Tandon, Suyoun Park, Thi Bich Ngoc Nguyen, Shinsuke Kawai*, Sung Ha Park**

S. Seo, T. H. N. Vu, A. Tandon, S. Park, T. B. N. Nguyen, Prof. S. H. Park

Department of Physics, Institute of Basic Science, and Sungkyunkwan Advanced Institute of Nanotechnology (SAINT), Sungkyunkwan University, Suwon 16419, Republic of Korea

E-mail: sunghapark@skku.edu

Prof. S. Kawai

Faculty of Science, Yamagata University, Yamagata 990-8560, Japan

E-mail: kawai@sci.kj.yamagata-u.ac.jp

**Supporting information includes Tables S1–S3, Figures S1, S2, S3 and descriptions of the quantitative comparisons between existing DNA coding algorithms and randomized DNA base sequence design rule R_N_-B#, convergence behavior of sequence randomness parameters under varying analysis coverage across DNA coding schemes, the mean square displacement, and the inverse Ising model.**

**Table S1.** Encoding rule table of randomized DNA base sequence design rule R_0_-B9.

**Table S2.** Encoding rule table of randomized DNA base sequence design rule R_∞_-B16.

**Table S3.** Encoding rule table of randomized DNA base sequence design rule R_1_-B12.

**S1. Quantitative Comparisons Between Existing DNA Coding Algorithms and Randomized DNA Base Sequence Design Rule R_N_-B#**

**Figure S1.** Comparison of DNA encoding schemes across various digital formats.

**Figure S2.** Comparison of polarization (γ) and interaction strength (λ) results across different binary matrix sizes.

**S2. Convergence Behavior of Sequence Randomness Parameters under Varying Analysis Coverage across DNA Coding Schemes**

**Figure S3.** Convergence behavior of sequence randomness parameters under varying analysis coverage across DNA coding schemes.

**S3. Mean Square Displacement**

**S4. Inverse Ising model**

| Previous  Unit | Next Number | | | | | | | | |
| --- | --- | --- | --- | --- | --- | --- | --- | --- | --- |
|  | 0 | 1 | 2 | 3 | 4 | 5 | 6 | 7 | 8 |
| TA | CA | CT | CG | GA | GT | GC | TA | TG | TC |
| GA | TC | CA | CT | CG | GA | GT | GC | TA | TG |
| CA | TG | TC | CA | CT | CG | GA | GT | GC | TA |
| AT | AT | TG | TC | CA | CT | CG | GA | GT | GC |
| GT | GC | AT | TG | TC | CA | CT | CG | GA | GT |
| CT | GT | GC | AT | TG | TC | CA | CT | CG | GA |
| AG | TA | TG | TC | AT | TG | TC | CA | CT | CG |
| TG | CG | TA | TG | TC | AT | TG | TC | CA | CT |
| CG | CT | CG | TA | TG | TC | AT | TG | TC | CA |
| AC | GA | GT | GC | TA | TG | TC | AT | TG | TC |
| TC | AC | GA | GT | GC | TA | TG | TC | AT | TG |
| GC | AG | AC | GA | GT | GC | TA | TG | TC | AT |

**Table S1.** Encoding rule table of randomized DNA base sequence design rule R_0_-B9.

| Previous  Unit | Next Number | | | | | | | | | | | | | | | |
| --- | --- | --- | --- | --- | --- | --- | --- | --- | --- | --- | --- | --- | --- | --- | --- | --- |
|  | 0 | 1 | 2 | 3 | 4 | 5 | 6 | 7 | 8 | 9 | a | b | c | d | e | f |
| AA | TA | TT | TG | TC | GA | GT | GG | GC | CA | CT | CG | CC | AA | AT | AG | AC |
| TA | AC | TA | TT | TG | TC | GA | GT | GG | GC | CA | CT | CG | CC | AA | AT | AG |
| GA | AG | AC | TA | TT | TG | TC | GA | GT | GG | GC | CA | CT | CG | CC | AA | AT |
| CA | AT | AG | AC | TA | TT | TG | TC | GA | GT | GG | GC | CA | CT | CG | CC | AA |
| AT | AA | AT | AG | AC | TA | TT | TG | TC | GA | GT | GG | GC | CA | CT | CG | CC |
| TT | CC | AA | AT | AG | AC | TA | TT | TG | TC | GA | GT | GG | GC | CA | CT | CG |
| GT | CG | CC | AA | AT | AG | AC | TA | TT | TG | TC | GA | GT | GG | GC | CA | CT |
| CT | CT | CG | CC | AA | AT | AG | AC | TA | TT | TG | TC | GA | GT | GG | GC | CA |
| AG | CA | CT | CG | CC | AA | AT | AG | AC | TA | TT | TG | TC | GA | GT | GG | GC |
| TG | GC | CA | CT | CG | CC | AA | AT | AG | AC | TA | TT | TG | TC | GA | GT | GG |
| GG | GG | GC | CA | CT | CG | CC | AA | AT | AG | AC | TA | TT | TG | TC | GA | GT |
| CG | GT | GG | GC | CA | CT | CG | CC | AA | AT | AG | AC | TA | TT | TG | TC | GA |
| AC | GA | GT | GG | GC | CA | CT | CG | CC | AA | AT | AG | AC | TA | TT | TG | TC |
| TC | TC | GA | GT | GG | GC | CA | CT | CG | CC | AA | AT | AG | AC | TA | TT | TG |
| GC | TG | TC | GA | GT | GG | GC | CA | CT | CG | CC | AA | AT | AG | AC | TA | TT |
| CC | TT | TG | TC | GA | GT | GG | GC | CA | CT | CG | CC | AA | AT | AG | AC | TA |

**Table S2.** Encoding rule table of randomized DNA base sequence design rule R_∞_-B16.

| Previous  Unit | Next Number | | | | | | | | | | | |
| --- | --- | --- | --- | --- | --- | --- | --- | --- | --- | --- | --- | --- |
|  | 0 | 1 | 2 | 3 | 4 | 5 | 6 | 7 | 8 | 9 | a | b |
| TA | AT | AG | AC | TA | TG | TC | GA | GT | GC | CA | CT | CG |
| GA | CG | AT | AG | AC | TA | TG | TC | GA | GT | GC | CA | CT |
| CA | CT | CG | AT | AG | AC | TA | TG | TC | GA | GT | GC | CA |
| AT | CA | CT | CG | AT | AG | AC | TA | TG | TC | GA | GT | GC |
| GT | GC | CA | CT | CG | AT | AG | AC | TA | TG | TC | GA | GT |
| CT | GT | GC | CA | CT | CG | AT | AG | AC | TA | TG | TC | GA |
| AG | GA | GT | GC | CA | CT | CG | AT | AG | AC | TA | TG | TC |
| TG | TC | GA | GT | GC | CA | CT | CG | AT | AG | AC | TA | TG |
| CG | TG | TC | GA | GT | GC | CA | CT | CG | AT | AG | AC | TA |
| AC | TA | TG | TC | GA | GT | GC | CA | CT | CG | AT | AG | AC |
| TC | AC | TA | TG | TC | GA | GT | GC | CA | CT | CG | AT | AG |
| GC | AG | AC | TA | TG | TC | GA | GT | GC | CA | CT | CG | AT |

**Table S3.** Encoding rule tables of randomized DNA base sequence design rule R_1_-B12.

**S1. Quantitative Comparisons Between Existing DNA Coding Algorithms and Randomized DNA Base Sequence Design Rule R_N_-B#**

Figure S1 presents representative digital files accompanied by quantitative evaluation results of encoded DNA sequences generated using conventional DNA coding algorithms (i.e., HEDGES, DNA Fountain, and yin-yang codec) and the randomized DNA base sequence design rule (i.e., R_∞_​-B16 and R_0_-B9).^[1-6]^ Figure S1(a) displays four different digital files with their respective file sizes: text document (On the Origin of Species.txt), classical music file (Allegro in C major.mp3), 763 × 763-pixel-size grayscale image (Landscape.png), and research article (Molecular Structure of Nucleic Acids.pdf). The first 3,000 bytes of each file are used as original data. Figure S1(b) shows the information density of DNA sequences encoded by six encoding schemes (i.e., Simple Mapping, HEDGES, DNA Fountain, yin–yang codec (YYC), R_∞_​-B16, and R_0_-B9). The HEDGES is configured with 512 bits per strand, maximum homopolymer length limit of 4-nt, and GC content range constrained between 0.4 and 0.6. The DNA Fountain is configured with a chunk size of 128 bytes (equivalent to 1,024 bits per strand), no Reed-Solomon parity bytes, maximum homopolymer length limit of 4-nt, and GC content range constrained between 0.4 and 0.6. The yin-yang codec (YYC) is configured with a maximum homopolymer length limit of 4-nt, and GC content range constrained between 0.4 and 0.6. Figure S1(c) shows GC ratio of DNA sequences encoded from each encoding scheme. GC ratio of 0.5 is marked with a red dot-line. An MP3 music data that have an unbalanced GC ratio (0.161) under simple mapping (00 → A, 01 → C, 10 → G, and 11 → T) exhibits more balanced GC ratio values (around 0.5) when encoded through other encoding schemes. Figure S1(d) represents maximum homopolymer length measured from each encoding scheme. Although long homopolymers are observed in simple mapping, which lacks sequence randomization (e.g., a 7,214-nt-long in MP3 data), other encoding schemes produce randomized sequences with considerably shorter homopolymer lengths. Even though the maximum homopolymer length is set to 4-nt in HEDGES, DNA Fountain, and yin-yang codec, homopolymers longer than 4-nt are observed in HEDGES, DNA Fountain, and yin-yang codec by joining multiple encoded strands together for analysis. Figure S1(e) displays DNA sequence randomness assessment results by using 2-dimensional active particle trajectory model. The velocity (V) and diffusion coefficients (D) distribution is shown across six encoding schemes for four digital files with different marks: circle for simple mapping, square for HEDGES, triangle for DNA Fountain, diamond for YYC, plus sign for R_∞_​-B16, and multiplication sign R_0_-B9. Figure S1(f) shows DNA sequence randomness assessment results through 3- dimensional active particle trajectory model. The angular velocity (ω) and rotational diffusion coefficient (D_R_​) distribution is displayed for six encoding schemes. Figure S1(g) DNA sequence represents randomness assessment results of a 3-input 1-output logic algorithm system model. The root-means-square values of parameters α and β (i.e., RMS(α) and RMS(β)) distribution is displayed. Across the three assessment models shown in Figure S1(e–g), DNA sequences encoded with R_0_-B9 exhibit consistently higher levels of randomization compared to those generated by other encoding schemes, independent of the characteristics of the original data.

[1] Y. Erlich, D. Zielinski, *Science* **2017**, 355, 950–954.

[2] W. H. Press, J. A. Hawkins, S. K. Jones, J. M. Schaub, I. J. Finkelstein, *Proc. Natl. Acad. Sci.* **2020**, 117, 18489–18496.

[3] Z. Ping, J. Chen, C. Xu, H. Li, Z. Zhou, M. Zhang, X. Chen, L. Xu, Y. Liu, Z. Zhang, C. Yuan, *Nature Comput. Sci.* **2022**, 2, 234–242.

[4] J. D. Watson, F. H. C. Crick, *Nature* **1953**, 171, 737. (PDF accessed at https://dosequis.colorado.edu/Courses/MethodsLogic/papers/WatsonCrick1953.pdf)

[5] W. A. Mozart, Allegro in C major, K.1b [MP3 audio file], Musopen, https://musopen.org/music/ (accessed Sep 26, 2025).

[6] C. Darwin, On the Origin of Species [electronic text], Project Gutenberg, https://www.gutenberg.org/files/1228/1228-h/1228-h.htm (accessed Sep 26, 2025).


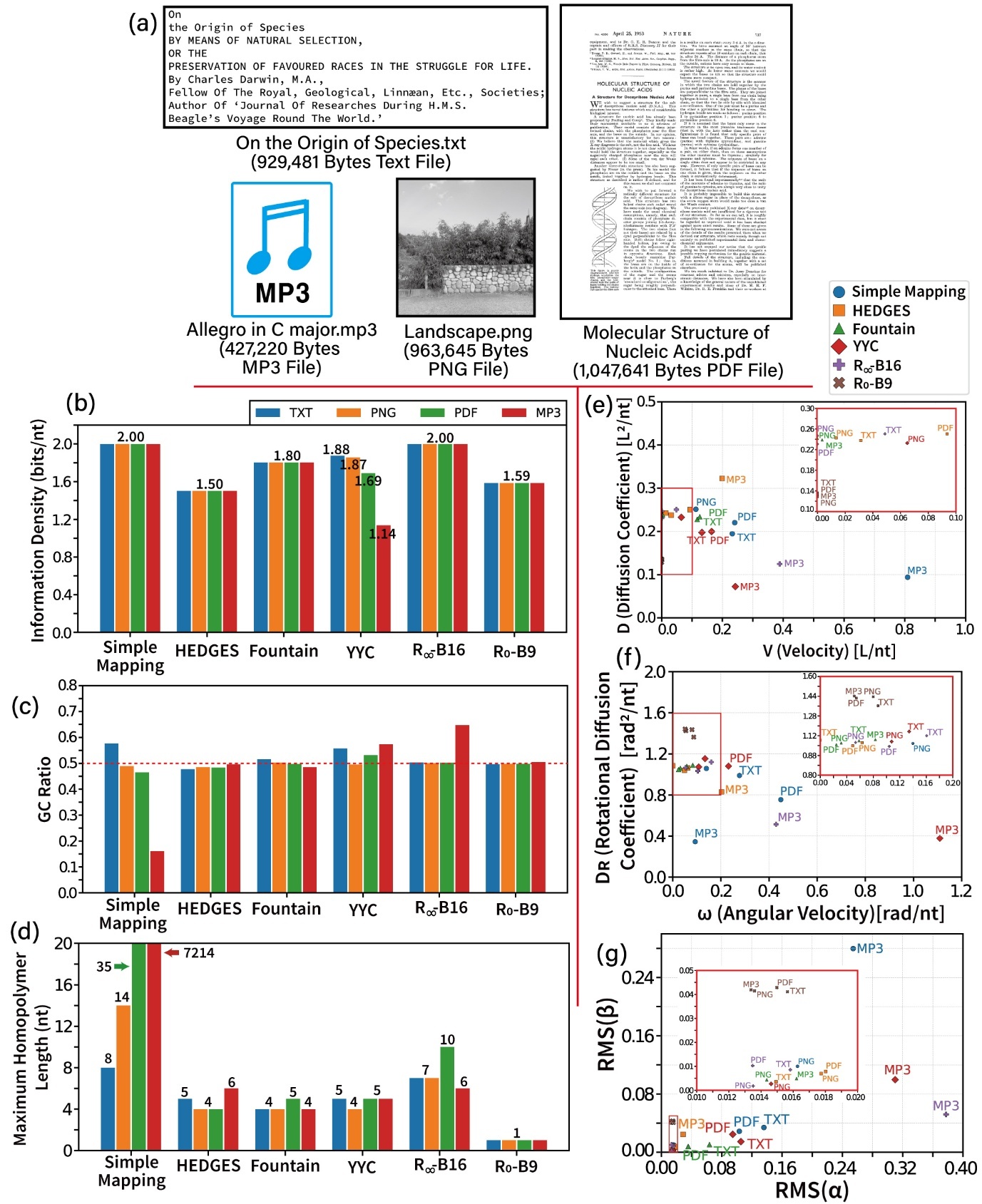


**Figure S1.** Comparison of DNA encoding schemes across various digital formats. (a) Representative digital files [i.e., text document (.txt), classical music file (.mp3), grayscale image (.png), and research article (.pdf)] used for encoding into DNA sequences. Only the first 3,000 bytes of each file are used as original input data. (b) Information density achieved by DNA coding schemes (i.e., Simple Mapping, HEDGES, DNA Fountain, yin–yang codec (YYC), R_∞_​-B16, and R_0_-B9). (c-d) Sequence characteristics of encoded DNA base sequences such as GC ratio and maximum homopolymer lengths. (e) Randomness assessment results of DNA sequences (across file formats) by using 2-dimensional active particle trajectory model. Velocity (V) and diffusion coefficients (D) are calculated from 12,000-nt sequences. (f) Randomness assessment results of DNA sequences through 3-dimensional active particle trajectory model. Angular velocity (ω) and rotational diffusion coefficient (D_R_​) are measured using 12,000-nt DNA sequences. (g) Comparison of root-means-square (RMS) values of parameters α and β derived from a 3-input 1-output logic algorithm system model.


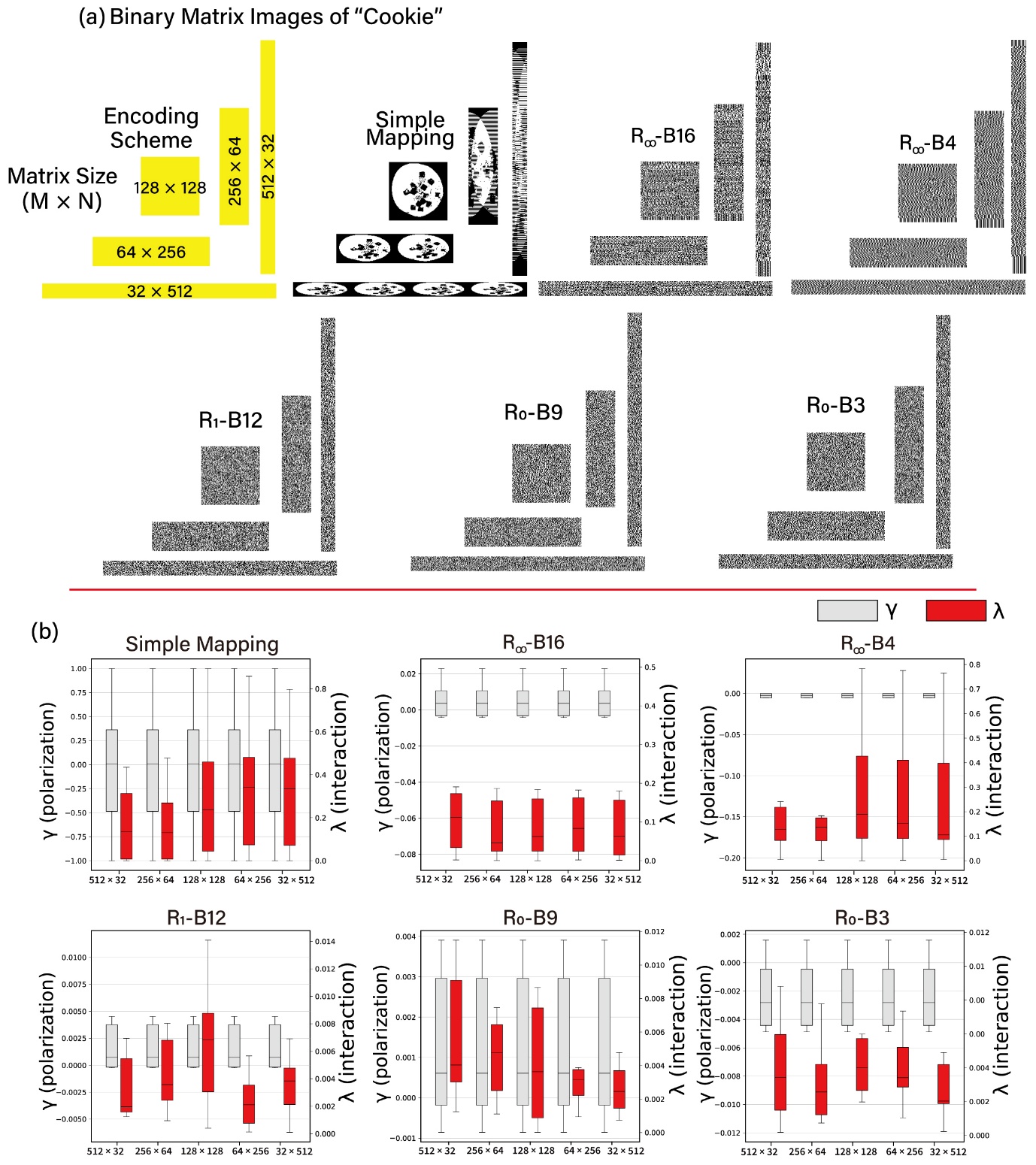


**Figure S2.** Comparison of polarization (γ) and interaction strength (λ) results across different binary matrix sizes. (a) Representative binary matrix images obtained by rearranging binary strings with different sizes (i.e., 512 × 32, 256 × 64, 128 × 128, 64 × 256, and 32 × 512). A binary string is created by mapping (A → 00, C → 01, G → 10, T → 11) DNA sequences into binary form. These nucleotide bases originate from sources such as the Cookie image, following a specific design rule (e.g., R_∞_-B16). (b) Quantitative analysis of encoding schemes as a function of binary matrix size using the inverse Ising model. Two parameters—polarization factor (γ) and interaction factor (λ)—are measured for all test images introduced in Figure 1(a), allowing assessment of whether the computed values are consistent across different lattice arrangements.

**S2. Convergence Behavior of Sequence Randomness Parameters under Varying Analysis Coverage across DNA Coding Schemes**

Figure S3 displays convergence behaviours of randomness assessment parameters (i.e., maximum homopolymer length, GC ratio, velocity V, diffusion coefficient D, angular velocity ω, and rotational diffusion coefficients D_R_) under varying analysis coverage (defined as the percentage of analysed sequence length among total encoded sequence length). Original “Cookie” image data (shown in Figure 1(a); 128 × 128 pixels, black and white) is encoded into 8,192-nt, 8,194-nt, 9,144-nt, and 10,340-nt length DNA sequences via simple mapping (SM; 00 to A, 01 to C, 10 to G, 11 to T), R_∞_-B16, R_1_-B12, and R_0_-B9, respectively. Figure S3 (a-b) shows the measured conventional sequence analysis parameters [i.e., maximum homopolymer length (HP) and GC ratio] as a function of analysis coverage percentage. The parameters HP and GC ratio are measured at every 10-nt interval. That is, the measurements are performed for 10-nt, 20-nt, 30-nt, and so on from the beginning of the encoded DNA sequence. Figure S3 (c-d) represents the sequence randomness assessment results using 2-dimensional active particle trajectory (2D-aPT) model (i.e., velocity V and diffusion coefficients D) with respect to adopted analysis coverage. Figure S3 (e-f) displays the assessment results via 3-dimensional active particle trajectory (3D-aPT) model (i.e., velocity ω and diffusion coefficients D_R_) as a function of analysis coverage. In 2D-aPT and 3D-aPT model, encoded sequences are assessed at every 20-nt interval.

Across all measured randomness parameters, strong fluctuations are observed at low analysis coverage (typically below 10% analysis coverage), representing that short read DNA sequence fragments are insufficient to represent the overall randomness of the entire encoded DNA sequence. When the analysis coverage over 50%, all measured parameters tend to converge stably without significant fluctuation, suggesting statistical representativeness of the entire encoded sequences.

Whereas the maximum homopolymer length is highly dependent on the local distribution of the encoded sequence (as evidenced by the large fluctuations at high analysis coverage observed in SM), the parameters derived from the 2D-aPT and 3D-aPT models (i.e., *V*, *D*, *ω*, and *D_R_*) exhibit markedly lower sensitivity to local sequence distribution, showing minimal variation in SM. This indicates the model’s robustness and representativeness as stable quantitative descriptors of sequence randomness. In contrast to the conventional indicators maximum homopolymer length and GC ratio, which exhibit low discriminative ability for sequence randomness (i.e., SM ≫ R_∞_-B16 ≈ R_1_-B12 ≈ R_0_-B9), the 2D-aPT and 3D-aPT models provide more continuous parameter distributions with wider resolution, representing superior reliability, and scalability for encoded DNA sequence randomness assessment.

In addition, the encoded sequence via SM exhibits relatively larger variations across all measured parameters with respect to analysis coverage compared to the encoded sequences through R_N_-B# (i.e., R_∞_-B16, R_1_-B12, and R_0_-B9) coding schemes. This indicates that the R_N_-B# coding schemes have less dependency on the local distribution of the original binary data and are capable of designing DNA sequences with globally uniform nucleotide composition.


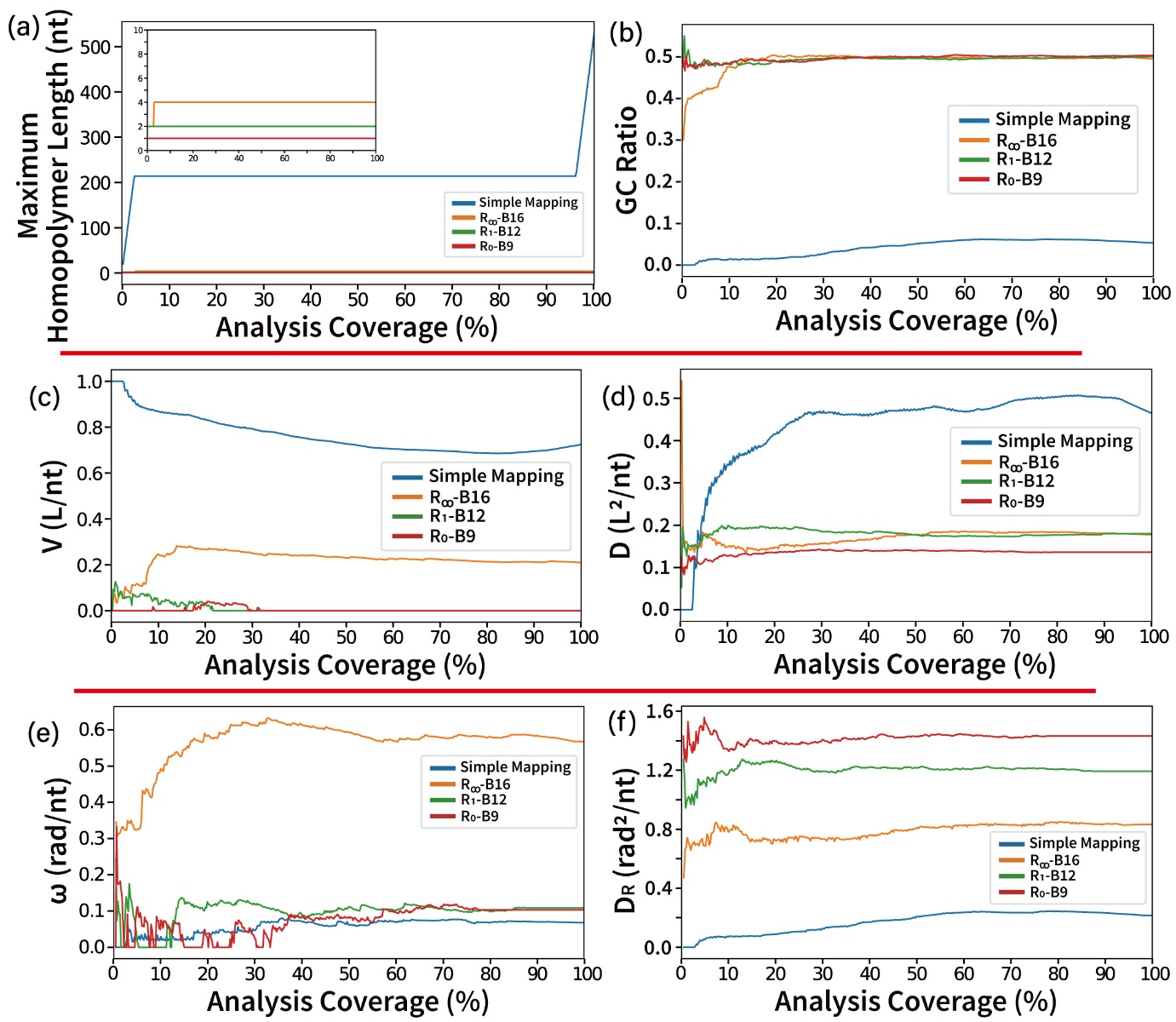


**Figure S3.** Convergence behavior of sequence randomness parameters (i.e., maximum homopolymer length, GC ratio, velocity *V*, diffusion coefficient *D*, angular velocity *ω*, and rotational diffusion coefficients *D_R_*) under varying analysis coverage (defined as the percentage of analyzed sequence length among total encoded sequence length) across DNA coding schemes [i.e., simple mapping (SM), R_∞_-B16, R_1_-B12, and R_0_-B9]. Original data is “Cookie” image (128 × 128 pixels, black and white) in Figure 1. Obtained DNA sequence length via SM, R_∞_-B16, R_1_-B12, and R_0_-B9 are 8,192-nt, 8,194-nt, 9,144-nt, 10,340-nt, respectively. (a) Maximum homopolymer length observed within the analyzed DNA sequence as a function of analysis coverage. (b) Measured GC ratio with respect to adopted analysis coverage percentage. (c-d) Measured sequence randomness assessment parameters (i.e., *V* [L/nt], and *D* [L^2^/nt]) with respect to analysis coverage under 2-dimensional active particle trajectory (2D-aPT) model. (e-f) Measured sequence randomness assessment parameters (i.e., *ω* [rad/nt], and *D_R_* [rad^2^/nt]) as a function of analysis coverage under 3-dimensional active particle trajectory (3D-aPT) model. (a-f) Measured parameter values for SM, R_∞_-B16, R_1_-B12, and R_0_-B9 are marked in blue, orange, green, and red lines, respectively.

**S3. Mean Square Displacement**

The mean square displacement of active particles is calculated using the equation below from Langevin mechanics.^[1]^

$$\left\langle{(\Delta r)}^{2} \right\rangle=4D\Delta n+ \frac{V^{2}{\tau_{R}}^{2}}{2}\left[ \frac{2\Delta n}{\tau_{R}}+e^{{-2\Delta n}/{\tau_{R}}}-1 \right]$$

Here, *D* is a diffusion coefficient, Δn is the time step number, V is the velocity (related with self-propelled motion), and τ_R_ is the inverse of the rotational diffusion coefficient (i.e., characteristic time). Over short time frames (Δn << τ_R_), the mean square displacement can be approximated as ˂(Δr)^2^˃ = 4DΔn + V^2^Δn^2^.

[1] J. R. Howse, R. A. Jones, A. J. Ryan, T. Gough, R. Vafabakhsh, R. Golestanian, *Phys. Rev. Lett.* **2007**, *99*, 048102.

**S4. Inverse Ising model**

The Ising model represents a system as a binary matrix of spins that can take values of +1 (up) or −1 (down). Each spin interacts with its neighbouring spins, and the overall behaviour of the system depends on these interactions. The Ising model with two-dimensional binary matrix data is defined by the following partition function, where the variable σ_i_ can have binary values of either +1 or −1 at the i^th^ node.^[1,2]^

$$Z_{(\lambda,h)}=\sum_{\sigma} e^{\lambda\sum_{\langle i,j\rangle} \sigma_{i}\sigma_{j}+h\sum_{i} \sigma_{i}}$$

The total number of nodes in the system is given by N_T_ = N × M, while the total number of node connections is calculated as N_n_ = (N − 1) × M + (M − 1) × N. Additionally, the total number of touching (i.e., nearest neighbour) node connections is determined as N_t_ = 12 × (N − 1) × (M − 1) – 4. Two sums are essential to compute the inverse Ising parameters,. The first is the summation over all spin states, given by Σ_i_ σ_i_ = (−1) × N₀ + (+1) × N₁, where N₀ and N₁ represent the numbers of 0-bits and 1-bits, respectively. The second key sum accounts for pairwise spin interactions among connected (i.e., nearest neighbour) nodes, expressed as Σ_i,j_ σ_i_σ_j_. With these parameters, the polarization factor is defined as γ(σ_i_, N_T_) = (Σ_i_ σ_i_) / N_T_. Similarly, the interaction factor λ(σ_i_ σ_j_, N_T_, N_n_, N_t_) and the bias factor *h*(σ_i_ σ_j_, N_T_, N_n_, N_t_) are computed as

$$\lambda=\frac{\epsilon+\gamma^{2}}{(\frac{N_{t}}{N_{n}}+1)\gamma^{4}+(\frac{4N_{n}}{N_{T}}(1-\gamma^{2})-\frac{N_{t}}{N_{n}})\gamma^{2}-1}$$

$$h=\frac{1}{2}\ln(\frac{1+\gamma}{1-\gamma})-\frac{2N_{n}\gamma\left( \epsilon+\gamma^{2} \right)}{N_{T}\left[ \left( \frac{N_{t}}{N_{n}}+1 \right)\gamma^{4}+\left( \frac{4N_{n}}{N_{T}}\left( 1-\gamma^{2} \right)-\frac{N_{t}}{N_{n}} \right)\gamma^{2}-1 \right]}$$

where ε ≡ (Σ_i,j_ σ_i_σ_j_) / N_n_.

[1] J. Strečka, M. Jaščur, *Acta Phys. Slovaca* **2015**, *65*, 235–367.

[2] H. C. Nguyen, R. Zecchina, J. Berg, *Adv. Phys.* **2017**, *66*, 197–261.
